# Dissecting Relations between Depression Severity, Antidepressant Use, and Metabolic Syndrome Components in the NHANES 2005–2020

**DOI:** 10.3390/jcm12123891

**Published:** 2023-06-07

**Authors:** Ziying Lin, Yap-Hang Chan, Bernard Man Yung Cheung

**Affiliations:** 1 Department of Medicine, School of Clinical Medicine, Li Ka Shing Faculty of Medicine, The University of Hong Kong, Queen Mary Hospital, 102 Pokfulam Road, Pokfulam, Hong Kong, China; 2 State Key Laboratory of Pharmaceutical Biotechnology, The University of Hong Kong, Pokfulam, Hong Kong, China; 3 Institute of Cardiovascular Science and Medicine, The University of Hong Kong, Pokfulam, Hong Kong, China

**Keywords:** metabolic syndrome components, depressive symptoms, antidepressant use, survey

## Abstract

We aimed to dissect the complex relations between depressive symptoms, antidepressant use, and constituent metabolic syndrome (MetS) components in a representative U.S. population sample. A total of 15,315 eligible participants were included from 2005 to March 2020. MetS components were defined as hypertension, elevated triglycerides, reduced high-density lipoprotein cholesterol, central obesity, and elevated blood glucose. Depressive symptoms were classified as mild, moderate, or severe. Logistic regression was used to evaluate the relationship between depression severity, antidepressant use, individual MetS components and their degree of clustering. Severe depression was associated with the number of MetS components in a graded fashion. ORs for severe depression ranged from 2.08 [95%CI, 1.29–3.37] to 3.35 [95%CI, 1.57–7.14] for one to five clustered components. Moderate depression was associated with hypertension, central obesity, raised triglyceride, and elevated blood glucose (OR = 1.37 [95%CI, 1.09–1.72], 1.82 [95%CI, 1.21–2.74], 1.63 [95%CI, 1.25–2.14], and 1.37 [95%CI, 1.05–1.79], respectively). Antidepressant use was associated with hypertension (OR = 1.40, 95%CI [1.14–1.72]), raised triglyceride (OR = 1.43, 95%CI [1.17–1.74]), and the presence of five MetS components (OR = 1.74, 95%CI [1.13–2.68]) after adjusting for depressive symptoms. The depression severity and antidepressant use were associated with individual MetS components and their graded clustering. Metabolic abnormalities in patients with depression need to be recognized and treated.

## 1. Introduction

Metabolic syndrome (MetS) is defined as a clustering of conditions that include hypertension, dyslipidemia (raised triglycerides and reduced high-density lipoprotein-cholesterol [HDL-C]), central obesity, and elevated fasting glucose. MetS is a key risk factor for cardiovascular diseases and is associated with increased all-cause mortality [1,2,3,4,5]. MetS as a major public health threat has been observed with increasing prevalence worldwide in recent decades [6].

Psychiatric conditions are associated with increased risks of premature death from cardiovascular diseases. Studies showed that an increased risk of MetS is present in psychiatric conditions, including depression [7,8,9]. Depression is now believed to be the result of an interaction of multiple risk factors, including genetics, development, and environment [10,11,12]. The convergence of evidence suggests that, despite the presence of multiple risk factors, the final pathologic pathway of depression occurs in multiple but interacting regions of the human brain, involving the prefrontal cortex and its connections to many subcortical structures [13,14,15,16]. Connections have been found between cortico-limbic circuits and shared physiological functions in chronic stress exposure and depression [17,18,19]. These processes involve disruptions in circadian rhythm function, affective state regulation, drive and motivation, intention formation, and behavioral execution. As a logical consequence of these disruptions, individuals may experience symptoms such as decreased energy levels, apathy, anhedonia, and changes in appetite and sleep patterns [12,20]. The unhealthy lifestyle and poor adherence to treatment associated with these changes may be potential mediating mechanisms between depressive patients and the presence of MetS [21,22]. Furthermore, underlying pathophysiological mechanisms, such as dysregulation of the hypothalamic–pituitary–adrenal (HPA) axis, impaired thalamocortical rhythm and immune function, and genetic predisposition, may have a long-term effect on depression and MetS in a bidirectional manner [23,24,25,26]. Thus, depression and MetS, both cardiovascular risk factors, may exert bidirectional influences through complex behavioral and psycho-neuroendocrine pathways. 

Although the link between depression and MetS has been established, the association between gradated depression severity and the individual components of MetS and the clustering of these components has not been well characterized [7,12]. Antidepressants may also directly influence metabolic dysregulation, independent of depressive symptoms [27]. Given that the MetS is a syndromal conglomeration of various metabolic abnormalities, describing its individual components and the clustering of its components may be a more robust way to determine the independent and synergistic effect between depression and MetS rather than using the dichotomous definition of MetS. Therefore, the present study aimed to dissect the complex associations between the severity of depressive symptoms, antidepressant use, and individual MetS components, as well as their degree of clustering in a representative non-institutionalized population sample, the U.S. National Health and Nutrition Examination Survey (NHANES).

## 2. Methods

### 2.1. Study Population

NHANES is a cross-sectional national survey designed to collect information on health and nutrition in the U.S. non-institutionalized civilian population [28]. It is a stratified, complex, multistage, probabilistic survey conducted continuously in two-year cycles since 1999. Specific weights are assigned to participants to account for their different sampling probabilities and non-response rates. NHANES was approved by the CDC Institutional Review Board, and all participants provided written informed consent. Depression was evaluated using the World Health Organization Composite International Diagnostic Interview (CIDI) in NHANES 1999–2004 and Patient Health Questionnaire-9 (PHQ-9) in NHANES 2005–2020, respectively [29,30]. In our study, we combined multiple years of NHANES data, from 2005 to March 2020, to yield the available NHANES sample size. We extracted the participants’ information, including demographic characteristics, examination results, prescription information, and laboratory tests.

### 2.2. Definitions of MetS Components, Depressive Symptoms, and Antidepressant Use

MetS components were defined according to the National Cholesterol Education Program’s Adult Treatment Panel III criteria: (1) hypertension: systolic blood pressure at least 130 mm Hg or diastolic blood pressure at least 85 mm Hg or taking hypertension medications; (2) raised triglyceride: triglyceride level greater than 150 mg/dL; (3) reduced HDL-C: HDL-C level less than 40 mg/dL in men or less than 50 mg/dL in women; (4) central obesity: waist circumference greater than 102 cm in men or 88 cm in women; (5) raised blood glucose: fasting plasma glucose level at least 100 mg/dL or taking diabetes medications [5]. MetS was defined as having at least three of the above five components [5].

Depressive symptoms were assessed using the Patient Health Questionnaire-9 (PHQ-9), the instrument most widely used in primary care settings to screen for depression [12,31]. It assesses symptoms of a major depressive episode in the past two weeks, with a score ranging from 0–27. Depression severity was classified as normal (0–4), mild (5–9), moderate (10–14), and severe (15–27) [31]. A score of 10 or above (moderate or severe depressive symptoms) is the standard cut-off for identifying possible major depression [32].

The information of antidepressant use was extracted from NHANES prescription information files [28]. The information on medication use was collected during household interviews. Participants were asked if they have taken medications in the past thirty days. The participants were asked to show containers of all drugs when they answered “yes” to the question or report the drug name if the containers were not available. All prescription medication data were processed and categorized using the Multum Lexicon Plus database. The category of “antidepressants” was listed in the second level category whose first level category was “psychotherapeutic agents” [33,34]. Antidepressant use in our study was defined as the use of at least one antidepressant agent in the prior thirty days. 

### 2.3. Definitions of Potential Confounders

Participants’ demographic and socioeconomic information were collected by the self-reported questionnaires. Participants’ age was stratified into three groups of 20–44 years old, 45–64 years old, and ≥65 years old. Race/ethnicity was classified into non-Hispanic Whites, non-Hispanic Blacks, Mexican Americans, other Hispanic, and other race. Education levels were categorized into four groups: less than high school, high school, some college, and college or higher. Family income-to-poverty ratio was categorized as less than 130%, 130–349%, and 350% or more. Health insurance was classified as uninsured and insured status. Marital status was categorized into three groups: married or living with partner, widowed or divorced or separated, and never married. The information on body mass index (BMI) was extracted from the examination data. Participants with a BMI of 25 kg/m^2^ or more were defined as overweight and over 30 kg/m^2^ as obese. The lifestyle factors, including physical activity, smoking status, and alcohol consumption were defined. Physical activity was collected during a household interview. Moderate activity was defined as work or recreational activity that resulted in a slight increase in breathing or heart rate for at least ten minutes. Vigorous activity was defined as activity that resulted in a large increase in breathing or heart rate for at least ten consecutive minutes. Participants who have smoked 100 cigarettes in their lifetimes and currently smoke cigarettes every day or some days were defined as current smokers. Those who have ever smoked 100 or more cigarettes or not and do not smoke now were defined as former or never smokers. Alcohol consumption was classified according to self-reported average daily numbers of standard drinks. Moderate drinkers were defined as drinking fewer than four alcoholic drinks per day for women or five alcoholic drinks per day for men. Heavy drinkers were defined as having four or more drinks a day for women or five or more drinks a day for men.

### 2.4. Statistical Analysis

All analyses were performed accounting for NHANES’ complex survey design, including appropriate NHANES sampling weights, strata, and primary sampling units, as these factors affect the precision of the estimates. All estimates and 95% confidence intervals (CIs) were weighted to be nationally representative. Participants’ characteristics were compared using adjusted Pearson’s chi-squared test.

Binomial logistic regression was used to evaluate the association between depressive symptoms and antidepressant use with individual MetS components among all participants. Multinomial logistic regression was used to evaluate the association between depressive symptoms and antidepressant use with the number of clustered MetS components among all participants. Models were adjusted for the potential confounders. Models for sensitivity analyses were additionally adjusted for antidepressant use or depressive symptoms. Because the parallel assumption was not met in the ordinal logistic regression models, multinomial logistic regression models were used to test the odds ratios of groups with more MetS components to the group without MetS components by the severity of depressive symptoms and antidepressant use. The association was reported as odds ratio (OR) and 95%CIs. Statistical significance was assessed at 2-sided *p* values < 0.05. All analyses were performed using SPSS 26.0 (IBM Corp., Armonk, NY, USA).

## 3. Results

A total of 15,315 participants were included in the final studied population (equivalent to approximately 200 million U.S. adults). Amongst them, 15.4%, 4.6%, and 2.3% presented with mild, moderate, and severe depressive symptoms during the study period, respectively. Of all included participants, 82.9% had one or more MetS components, and more than 50% had central obesity and elevated blood glucose. 

**Figure 1:**
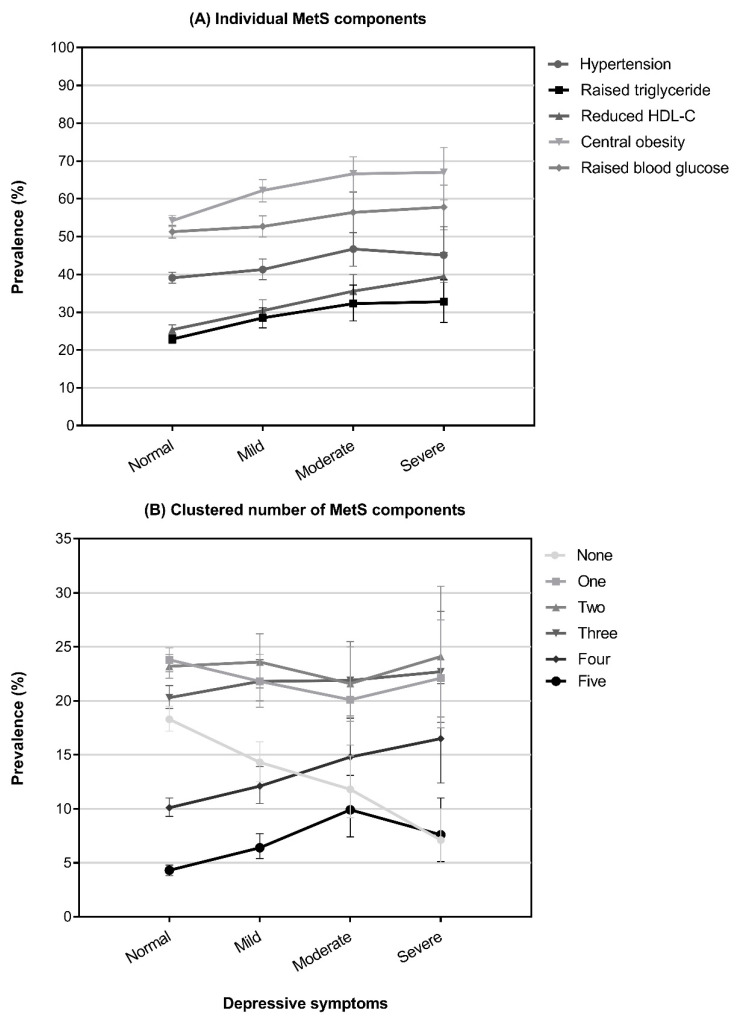
Prevalence of individual (**A**) and clustered (**B**) MetS components by depressive symptoms.

Baseline characteristics of all participants by individual MetS component and according to the degree of clustering are shown in Table 1, Table 2, and Table S1, respectively. Prevalence estimates of individual MetS components (A) and their clustered presence (B) according to depression severity are shown in Figure 1. Prevalence estimates for all five subgroups of abnormal components increased with depression severity. The proportions of participants with any depressive symptom amongst those with hypertension, raised triglyceride, reduced HDL-C and central obesity were significantly higher than those in the corresponding normal subgroups. This was not the case in those with raised blood glucose. More participants used antidepressants in these five subgroups compared to corresponding normal subgroups. The prevalence of depressive symptoms and antidepressants increased with the number of MetS components in an individual (*p* < 0.001). More women than men experienced depressive symptoms, whether mild (18.0% vs. 12.8%), moderate (6.0% vs. 3.3%), or severe (2.8% vs. 1.8%) (Table S2). More than twice as many women, compared to men, used antidepressants (17.6% vs. 7.4%) (Table S2). In each of the men and women subgroups, there was a significant positive association between the severity of depression and the number of MetS components (Table S3). 

As shown by multivariable models in Table 3, Tables S4 and S5, compared with subjects without depression, those with depression had a higher risk of developing raised triglyceride: ORs for mild, moderate, and severe depression were 1.30 (95%CI [1.12–1.52]), 1.63 (95%CI [1.25–2.14]), and 1.44 (95%CI [1.05–1.97]), respectively. Those with moderate depression were more likely to have hypertension (OR = 1.37 [95%CI, 1.09–1.72]), central obesity (OR = 1.82 [95%CI, 1.21–2.74]) and raised blood glucose (OR = 1.37 [95%CI, 1.05–1.79]) (Table 3). Interestingly, the association between moderate depression and hypertension was not statistically significant after adjustment for antidepressant use (Table S4, Model 2). Furthermore, increased depression severity was associated with increased clustering of MetS components in a graded fashion (Table 3). Compared with subjects without depression, those with mild depression had a higher risk of having five MetS components (OR = 1.82 [95%CI, 1.25–2.67], versus none). Similarly, those with moderate and severe depression had more than 4-fold (OR = 4.31 [95%CI, 2.45–7.60]) and 3-fold risk (OR = 3.35 [95%CI, 1.57–7.14]) of having five MetS components, respectively. These findings remained similar after adjustment for antidepressant use. Gender-specific analyses are shown in Figure S1 and Table S8.

**Figure 2:**
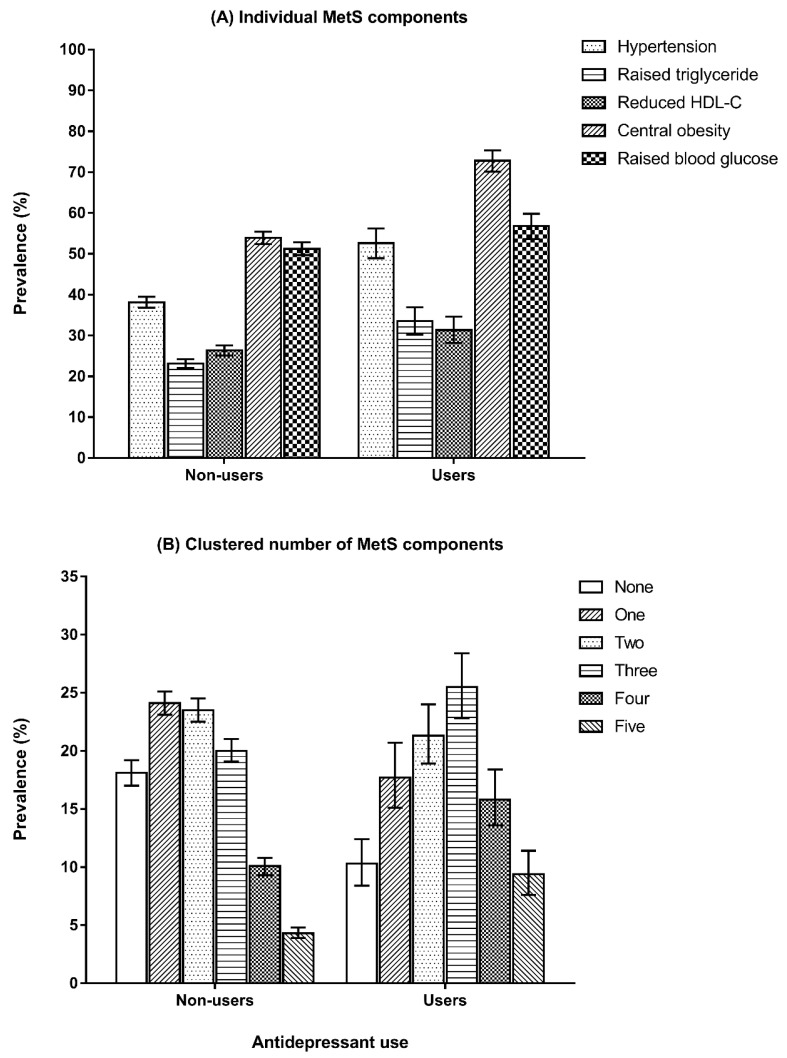
Prevalence of individual (**A**) and clustered (**B**) MetS components by antidepression use.

Prevalence estimates of individual MetS components (A) and their clustered presence (B) stratified by antidepressant use are shown in Figure 2. The prevalence of each individual MetS component was higher amongst antidepressant users compared to non-users. Independent of the severity of depression, antidepressant use was associated with hypertension (OR = 1.40 [95%CI, 1.14–1.72]) and raised triglyceride (OR = 1.43 [95%CI, 1.17–1.74]) (Table 4, Tables S6 and S7). Such associations were not statistically significant for HDL-C, central obesity, and elevated blood glucose. Furthermore, antidepressant use was associated with the degree of clustering of MetS components (five MetS components versus none: OR = 1.74 [95%CI, 1.13–2.68]). Gender-specific analyses are shown in Figure S2 and Table S9. 

## 4. Discussion

Our study provides a comprehensive evaluation of the association between depression severity and antidepressant use with individual MetS components as well as their degree of clustering in the U.S. national survey over 15 years. Greater depression severity was not only associated with individual MetS components but their greater degree of clustering in a graded fashion. In addition, the use of antidepressants was further associated with greater degrees of clustering of MetS components, independently of the severity of depressive symptoms. It was also individually associated with higher risks of hypertension and elevated triglyceride.

Although relations between depression and MetS had been described earlier [7], the current study provides unique insights into the complex relations between gradated severity of depression and each constituent component of MetS among the non-institutionalized U.S. population. One previous study using the NHANES 2009–2010 dataset showed that 41% of depressed individuals (PHQ-9 score ≥ 10) fulfilled the criteria of MetS, which ties in with the estimate of 46% in our study [35]. Our findings indicate that persons with more severe depression could be at increased vulnerability to the clustering of MetS components. An excess two-fold increase in cardiovascular risk associated with MetS compared with the general population, including mortality, warrants a routine screening for depressed persons, especially those who have moderate or severe depression [2]. While the mechanism behind the association remains not entirely clear, depression has been reported to be intricately associated with MetS components through behavioral and psycho-neuroendocrine pathways. For example, depressive symptoms can affect individuals’ lifestyle behaviors, such as physical inactivity, heavy smoking, alcohol consumption, and unhealthy dietary patterns, thereby contributing to MetS development [22,36,37]. In addition, increasing studies suggest that the HPA axis hyperactivation is an inherent feature in different psychiatric patients that plays an important role by multiple paths, including increased glucocorticoid sensitivity and lipid storage, in the linkage between depression and MetS development [7,38,39]. Furthermore, systemic inflammatory stress has been implicated in depression, and inflammation is a cardinal metabolic derangement that is seen in MetS [25,35,40,41,42]. The activation of the HPA axis and inflammation response may affect the development of both disorders, and this influence could be long term [12,43]. Hence, clinicians need to adopt a holistic view of patients’ mental and physical health when managing depression, considering these interconnected factors in their treatment plans.

Interestingly, our findings showed a stronger association between the presence of individual MetS components and moderate depression, as compared to severe depression. Furthermore, antidepressant use was associated with an increase in certain MetS components, such as elevated triglyceride levels and hypertension, independent of depression severity. This might raise the possibility of a distinct role played by antidepressants, independent of their impact on depressive symptoms, in modulating certain MetS components. For instance, tricyclic antidepressants (TCAs) and serotonin and norepinephrine reuptake inhibitors (SNRIs) have been associated with hypertension and abdominal obesity, while selective serotonin reuptake inhibitors (SSRIs) have been shown to be associated with increased weight and dyslipidemia [44,45,46,47]. Antidepressants were shown to have pleiotropic effects across biological systems on processes such as immunity, oxidation, and inflammation [48,49]. Studies found that antidepressant treatment improved glycemic control in those patients with diabetes [50]. However, there is no evidence of increased or decreased cardiac events resulting from antidepressant use in meta-analyses or large randomized trials (sample size > 200 participants) [51,52,53]. Whether there is a genuine detrimental effect of antidepressants on MetS is uncertain. Alternatively, it is possible that the excess risks observed in MetS components and their clustering associated with antidepressant use in our study could have reflected a sample with higher pre-treatment depression severity that mandated antidepressant treatment. In fact, the abolishment of a positive association between depression severity and hypertension by including antidepressant use in the multivariable model may indicate potential protection of effective antidepressant therapy by ameliorating the harmful effects of depression on MetS. Thus, it is crucial for healthcare providers to closely monitor metabolic indicators in patients undergoing antidepressant treatment. Regular screening for MetS components and tailored interventions might be required to counteract potential adverse metabolic effects of antidepressants. 

Interestingly, our results showed potential gender differences in the association between depressive symptoms and antidepressant use with MetS. Studies have consistently found that depression is more common in women than men, as was the case in our study [54,55]. The potential behavioral and biological mechanisms involved in the sex differences for developing individual MetS components and their combination ought to be further investigated in future studies. 

### Strengths and Limitations

Our study, drawing on the recent 15 years of nationally representative U.S. data, emphasizes the need for healthcare providers to recognize the complex relationship between depression severity, antidepressant use, and individual MetS components and the degree of their clustering. Moreover, integrated care models that address both mental and physical health conditions concurrently might be beneficial in managing this dual burden of disease. Nevertheless, there were several important limitations. First, we could not infer direct causality based on the current results due to the cross-sectional nature. Second, the study data were also subject to response bias, as participants self-reported medication use. Medication compliance was not assessed. Third, our study did not delve into the potential effects of different categories of antidepressants on MetS components. Future research could benefit from a more nuanced exploration of this aspect, examining the differential metabolic impacts of various antidepressant classes and their potential underlying mechanism. Lastly, self-reported depressive symptoms may subject to response bias, and they may differ from the clinical features of depression assessed by a medical doctor. Future research to corroborate our findings is needed.

## 5. Conclusions

The severity of depressive symptoms was associated with individual MetS components and their clustering in the U.S. general population. The findings highlight the importance of an individualized approach in the management of cardiometabolic risk in patients with depression. Whether antidepressant treatment may result in tangible impacts on MetS in depressive persons will require further investigation.

## Figures and Tables

**Table 1:** Characteristics of participants by individual MetS components ^a^.

Characteristic	Total (*n* = 15,315)	Hypertension	*p* Value	Raised Triglyceride	*p* Value	Reduced HDL-C	*p* Value	Central Obesity	*p* Value	Raised Blood Glucose	*p* Value
		(*n*= 6952, 40.0 [38.7–41.3])		(*n* = 3783, 24.4 [23.4–25.4])		(*n* = 4259, 27.0 [25.8–28.2])		(*n* = 8780, 56.3 [54.9–57.7])		(*n* = 8493, 51.9 [50.5–53.4])	
**Age, years**			<0.001		<0.001		<0.001		<0.001		<0.001
20–44	45.5 (44.0–47.0)	20.1 (18.8–21.6)		39.1 (36.6–41.6)		49.2 (47.1–51.2)		37.5 (35.9–39.2)		31.9 (30.1–33.7)	
45–64	36.4 (35.2–37.6)	46.1 (44.6–47.7)		41.7 (39.3–44.1)		35.7 (33.8–37.6)		39.9 (38.6–41.3)		42.7 (41.1–44.3)	
≥65	18.2 (17.2–19.2)	33.7 (32.1–35.4)		19.2 (17.7–20.9)		15.1 (13.7–16.7)		22.5 (21.3–23.8)		25.5 (24.1–26.8)	
**Women**	50.6 (49.7–51.6)	48.3 (46.9–49.7)	<0.001	43.0 (40.9–45.0)	<0.001	54.5 (52.6–56.5)	<0.001	60.0 (58.6–61.5)	<0.001	43.0 (41.6–44.5)	<0.001
**Race/ethnicity**			<0.001		<0.001		<0.001		<0.001		<0.001
Non-Hispanic White	67.8 (65.4–70.1)	69.1 (66.2–71.9)		71.5 (68.8–74.0)		66.7 (63.7–69.6)		69.8 (67.1–72.4)		67.8 (65.3–70.3)	
Non-Hispanic Black	10.8 (9.6–12.2)	14.0 (12.2–16.0)		5.2 (4.4–6.2)		9.1 (7.8–10.6)		11.3 (9.9–13.0)		9.8 (8.6–11.1)	
Mexican American	8.4 (7.2–9.7)	5.8 (4.8–7.0)		10.2 (8.7–11.9)		10.0 (8.5–11.7)		8.4 (7.1–10.0)		9.1 (7.8–10.7)	
Other Hispanic	5.7 (4.8–6.6)	4.6 (3.8–5.6)		5.7 (4.5–7.1)		6.9 (5.8–8.2)		5.3 (4.5–6.3)		5.7 (4.8–6.7)	
Other race	7.3 (6.6–8.2)	6.5 (5.6–7.5)		7.4 (6.4–8.6)		7.3 (6.2–8.6)		5.1 (4.4–5.9)		7.6 (6.7–8.6)	
**Education**			<0.001		<0.001		<0.001		<0.001		<0.001
<High school	15.1 (14.0–16.3)	17.3 (16.0–18.7)		18.3 (16.7–20.0)		18.9 (17.3–20.6)		15.7 (14.5–17.0)		17.4 (16.1–18.8)	
High school	23.5 (22.3–24.8)	27 (25.4–28.8)		25.1 (22.9–27.5)		24.7 (22.6–26.9)		24.6 (23.3–26.0)		25.6 (24.1–27.2)	
Some college	31.0 (29.8–32.3)	31 (29.1–32.9)		32.1 (30.0–34.3)		33.0 (30.8–35.2)		33.3 (31.8–34.9)		29.6 (28–31.4)	
College or higher	30.4 (28.4–32.4)	24.7 (22.6–26.9)		24.5 (22.1–27.1)		23.4 (21.2–25.9)		26.3 (24.3–28.5)		27.3 (25.3–29.5)	
**Family income-to-poverty ratio**			0.05		0.09		<0.001		<0.001		0.16
<130%	20.0 (18.6–21.4)	19.3 (17.7–21.1)		21.2 (19.4–23.2)		25.2 (23.3–27.2)		20.4 (18.9–22.0)		19.8 (18.4–21.3)	
130–349%	36.1 (34.8–37.5)	37.7 (35.9–39.5)		36.8 (34.7–38.9)		38.5 (36.4–40.7)		38.0 (36.1–39.8)		37.2 (35.5–38.9)	
≥350%	43.9 (42.0–45.9)	42.9 (40.6–45.4)		42.0 (39.6–44.5)		36.3 (33.3–39.4)		41.6 (39.3–44.0)		43.0 (40.8–45.3)	
**Health insurance**	82.9 (81.7–84.0)	88.0 (86.8–89.1)	<0.001	82.5 (80.5–84.3)	0.54	80.0 (78.3–81.7)	<0.001	85.7 (84.4–86.9)	<0.001	84.8 (83.6–86.0)	<0.001
**Marital status**			<0.001		<0.001		0.54		<0.001		<0.001
Married/Living with Partner	64.3 (62.6–65.9)	65.3 (63.3–67.2)		67.3 (64.7–69.9)		64.5 (62.0–67.0)		65.7 (63.6–67.8)		66.7 (64.7–68.7)	
Widowed/Divorced/Separated	17.7 (16.7–18.7)	24.3 (22.7–25.9)		19.1 (17.4–21.0)		18.1 (16.6–19.7)		20.6 (19.2–22.0)		20.7 (19.2–22.2)	
Never married	18.1 (16.8–19.4)	10.5 (9.5–11.6)		13.5 (11.8–15.4)		17.3 (15.4–19.4)		13.7 (12.4–15.0)		12.6 (11.3–14.1)	
**BMI, kg/m^2^**			<0.001		<0.001		<0.001		<0.001		<0.001
Normal (<25)	29.6 (28.5–30.7)	18.7 (17.6–19.9)		13.0 (11.6–14.4)		13.7 (12.3–15.3)		4.8 (4.1–5.6)		18.4 (17.3–19.6)	
Overweight (25–<30)	33.2 (32.4–34.1)	32.3 (30.9–33.8)		35.4 (33.6–37.2)		30.6 (28.7–32.6)		31.3 (29.9–32.7)		34.0 (32.8–35.3)	
Obese (≥30)	37.2 (36.1–38.4)	49.0 (47.3–50.6)		51.6 (49.5–53.7)		55.7 (53.4–58.0)		63.9 (62.4–65.4)		47.6 (46.1–49.1)	
**Physical activity**			<0.001		0.04		0.17		<0.001		0.17
Little/None	49.9 (48.5–51.3)	52.5 (50.7–54.3)		48.3 (46.0–50.7)		51.3 (49.3–53.4)		51.6 (50.1–53.2)		50.6 (48.9–52.3)	
Moderate	25.3 (24.2–26.4)	26.1 (24.5–27.7)		27.3 (25.2–29.6)		25.0 (23.1–27.1)		26.4 (25.1–27.7)		25.4 (24.0–26.9)	
Vigorous	24.9 (23.8–26)	21.4 (20–22.9)		24.3 (22.5–26.3)		23.6 (21.9–25.5)		22.0 (20.7–23.2)		24.0 (22.7–25.4)	
**Current smoking**	19.7 (18.5–20.8)	17.3 (16.0–18.7)	<0.001	22.7 (21–24.5)	<0.001	24.8 (22.9–26.8)	<0.001	17.2 (16.0–18.5)	<0.001	18.4 (17.2–19.8)	0.01
**Alcohol consumption**			<0.001		<0.001		<0.001		<0.001		<0.001
Non-drinker	21.3 (20.1–22.6)	26.8 (25.2–28.5)		23.5 (21.7–25.4)		26.5 (24.6–28.5)		24.8 (23.3–26.4)		23.3 (21.8–24.8)	
Moderate drinker	62.8 (61.1–64.4)	59.4 (57.2–61.5)		57.6 (54.9–60.2)		55.5 (53.2–57.9)		60.0 (58.1–61.8)		61.7 (59.8–63.6)	
Heavy drinker	15.9 (15–16.8)	13.8 (12.6–15.2)		18.9 (17.0–20.9)		18.0 (16.4–19.7)		15.2 (14.2–16.4)		15.0 (13.8–16.3)	
**Depressive symptoms**			0.01		<0.001		<0.001		<0.001		0.08
Normal	77.6 (76.6–78.6)	76.0 (74.5–77.5)		72.7 (70.7–74.7)		73.1 (71–75.2)		74.7 (73.3–76.0)		76.7 (75.2–78.2)	
Mild	15.4 (14.6–16.3)	16.0 (14.7–17.3)		18.0 (16.4–19.8)		17.4 (15.6–19.3)		17.1 (15.9–18.3)		15.7 (14.5–17.0)	
Moderate	4.6 (4.2–5.1)	5.4 (4.7–6.2)		6.1 (5.2–7.3)		6.1 (5.3–7.1)		5.5 (4.9–6.2)		5.0 (4.4–5.7)	
Severe	2.3 (2–2.6)	2.6 (2.1–3.2)		3.1 (2.5–3.8)		3.4 (2.7–4.1)		2.7 (2.4–3.2)		2.6 (2.2–3.0)	
**Antidepressants use**	12.6 (11.8–13.4)	16.5 (15.4–17.8)	<0.001	17.2 (15.3–19.3)	<0.001	14.6 (13–16.3)	0.003	16.3 (15.2–17.4)	<0.001	13.7 (12.8–14.8)	<0.001

Abbreviations: BMI, body mass index; HDL-C, high-density lipoprotein-cholesterol; MetS, metabolic syndrome. ^a^ Prevalence estimates [% (95%CI)] are weighted to be nationally representative.

**Table 2:** Characteristics of participants by the clustered number of MetS components ^a^.

Characteristic	Total (*n* = 15,315)	None	One	Two	Three	Four	Five	*p* Value
		(*n* = 2230, 17.1 [16.1–18.2])	(*n* = 3322, 23.3 [22.3–24.3])	(*n* = 3701, 23.2 [22.2–24.2])	(*n* = 3483, 20.7 [19.8–21.6])	(*n* = 1801, 10.8 [10.1–11.6])	(*n* = 778, 4.9 [4.5–5.4])	
**Age, years**								
20–44	45.5 (44.0–47.0)	71.9 (69.4–74.3)	56.8 (54.4–59.1)	40.8 (38.2–43.5)	32.0 (29.6–34.4)	26.5 (23.9–29.3)	20.6 (16.8–24.9)	<0.001
45–64	36.4 (35.2–37.6)	23.7 (21.4–26.1)	31.9 (29.7–34.2)	39.0 (36.6–41.4)	40.3 (38.3–42.4)	47.6 (44.6–50.6)	47.8 (43.5–52.1)	
≥65	18.2 (17.2–19.2)	4.4 (3.5–5.6)	11.3 (9.8–13)	20.2 (18.4–22.1)	27.7 (25.5–30)	25.9 (23.9–27.9)	31.6 (27.7–35.8)	
**Women**	50.6 (49.7–51.6)	53.7 (50.8–56.5)	48.8 (46.3–51.4)	50.6 (48.4–52.8)	49.3 (47.1–51.5)	50.5 (46.6–54.4)	54.3 (50.0–58.6)	0.08
**Race/ethnicity**								
Non-Hispanic White	67.8 (65.4–70.1)	68.5 (65.7–71.2)	65.7 (62.6–68.7)	65.8 (62.8–68.7)	68.1 (64.9–71.2)	70.2 (66.8–73.4)	77.6 (73.6–81.2)	<0.001
Non-Hispanic Black	10.8 (9.6–12.2)	10.3 (8.9–11.8)	11.1 (9.6–12.9)	11.9 (10.4–13.6)	12.0 (10.4–14)	8.7 (7.3–10.4)	5.7 (4.3–7.6)	
Mexican American	8.4 (7.2–9.7)	7.0 (5.9–8.3)	8.8 (7.4–10.5)	9.0 (7.5–10.8)	8.5 (7.2–10.0)	8.8 (7.1–10.8)	6.9 (5.2–9.2)	
Other Hispanic	5.7 (4.8–6.6)	5.4 (4.2–6.8)	6.3 (5.2–7.6)	6.0 (5.0–7.2)	5.1 (4.2–6.1)	5.3 (4.3–6.6)	5.4 (3.8–7.6)	
Other race	7.3 (6.6–8.2)	8.9 (7.5–10.5)	8.0 (6.9–9.3)	7.3 (6.2–8.5)	6.3 (5.2–7.6)	6.9 (5.5–8.7)	4.3 (2.6–7.2)	
**Education**								
<High school	15.1 (14.0–16.3)	9.6 (8.1–11.3)	13.2 (11.6–15.0)	16.5 (14.9–18.1)	17.7 (16.0–19.6)	17.5 (15.4–19.9)	20.7 (17.9–23.9)	<0.001
High school	23.5 (22.3–24.8)	18.0 (15.8–20.4)	22.3 (20.1–24.7)	23.8 (21.6–26.2)	26.5 (24.5–28.5)	27.9 (24.7–31.3)	24.4 (21.1–28.1)	
Some college	31.0 (29.8–32.3)	30.4 (27.7–33.1)	29.9 (27.7–32.1)	30.9 (28.9–33)	31.7 (29.7–33.9)	32.3 (28.9–35.9)	33.8 (29.3–38.7)	
College or higher	30.4 (28.4–32.4)	42.1 (38.8–45.5)	34.6 (31.8–37.6)	28.8 (26–31.7)	24.1 (21.9–26.4)	22.3 (19.2–25.8)	21 (16.9–25.8)	
**Family income-to-poverty ratio**								
<130%	20.0 (18.6–21.4)	18.1 (16.3–20.0)	19.2 (17.2–21.3)	19.7 (17.8–21.7)	22.0 (19.8–24.5)	20.8 (18.3–23.5)	21.4 (18.4–24.7)	<0.001
130–349%	36.1 (34.8–37.5)	31.1 (28.4–33.8)	35.8 (33.6–38.0)	37.1 (34.8–39.5)	37.8 (35.2–40.6)	39.2 (36.1–42.5)	37.0 (32.7–41.5)	
≥350%	43.9 (42.0–45.9)	50.9 (47.5–54.2)	45.1 (42.5–47.7)	43.2 (40.2–46.2)	40.1 (36.9–43.5)	40.0 (36.4–43.7)	41.6 (36.6–46.8)	
**Health insurance**	82.9 (81.7–84)	79.5 (76.9–81.8)	80.8 (78.8–82.6)	82.4 (80.5–84.2)	85.7 (84.0–87.2)	85.7 (83.6–87.6)	89.1 (86.5–91.2)	<0.001
**Marital status**								
Married/Living with Partner	64.3 (62.6–65.9)	58.3 (55.6–61.0)	64.1 (61.6–66.5)	65.2 (62.6–67.8)	66.7 (64.1–69.3)	66.4 (62.5–70.0)	66.1 (61.4–70.4)	<0.001
Widowed/Divorced/Separated	17.7 (16.7–18.7)	9.5 (8.2–11.0)	15.1 (13.5–16.8)	18.8 (17.1–20.6)	21.9 (20.1–23.9)	23.1 (20.2–26.3)	23.4 (20–27.3)	
Never married	18.1 (16.8–19.4)	32.1 (29.6–34.8)	20.8 (18.6–23.2)	16.0 (14.1–18.0)	11.3 (9.9–12.9)	10.5 (8.6–12.9)	10.5 (8.0–13.8)	
**BMI, kg/m^2^**								
Normal (<25)	29.6 (28.5–30.7)	74.8 (72.5–77.0)	41.8 (39.6–44.0)	20.6 (19.0–22.2)	8.3 (7.1–9.8)	4.0 (3.1–5.0)	1.8 (1.1–3.0)	<0.001
Overweight (25–<30)	33.2 (32.4–34.1)	23.3 (21.2–25.6)	38.6 (36.6–40.6)	41.7 (39.2–44.1)	32.4 (30.4–34.5)	26.0 (23.4–28.8)	22.0 (18.4–26.2)	
Obese (≥30)	37.2 (36.1–38.4)	1.8 (1.3–2.7)	19.6 (17.8–21.5)	37.8 (35.5–40.2)	59.3 (56.7–61.8)	70.0 (67.3–72.7)	76.2 (72–79.9)	
**Physical activity**								
Little/None	49.9 (48.5–51.3)	48.4 (45.4–51.4)	47.4 (45.1–49.7)	50.9 (48.5–53.3)	50.5 (48.2–52.9)	50.7 (47.3–54.0)	57.1 (52.3–61.8)	<0.001
Moderate	25.3 (24.2–26.4)	23.9 (21.9–26.1)	24.2 (22.2–26.3)	25.1 (23.0–27.3)	26.2 (24.1–28.5)	28.8 (26.2–31.5)	23.9 (19.7–28.7)	
Vigorous	24.9 (23.8–26)	27.7 (25.4–30.2)	28.4 (26.0–31.0)	24.0 (21.9–26.2)	23.2 (21.3–25.3)	20.6 (18.0–23.4)	19.0 (15.6–22.9)	
**Current smoking**	19.7 (18.5–20.8)	20.4 (18.3–22.6)	20.3 (18.4–22.3)	19.6 (17.7–21.8)	19.2 (17.5–21.0)	18.6 (16.1–21.3)	18.8 (15.8–22.2)	0.80
**Alcohol consumption**								
Non-drinker	21.3 (20.1–22.6)	15.4 (13.3–17.7)	16.4 (14.9–18.1)	22.0 (20.3–23.8)	24.6 (22.5–26.9)	27.7 (25.1–30.5)	33.9 (30.0–38.1)	<0.001
Moderate drinker	62.8 (61.1–64.4)	68.9 (65.8–71.7)	67.5 (65.2–69.7)	62.2 (60.0–64.3)	58.0 (55.2–60.7)	57.2 (53.6–60.8)	53.9 (49.0–58.8)	
Heavy drinker	15.9 (15–16.8)	15.7 (14.0–17.7)	16.1 (14.4–17.9)	15.8 (14.2–17.5)	17.4 (15.5–19.4)	15.1 (12.8–17.7)	12.1 (9.4–15.6)	
**Depressive symptoms**								
Normal	77.6 (76.6–78.6)	83.0 (81.1–84.8)	79.4 (77.4–81.3)	77.6 (75.6–79.5)	76.3 (74.2–78.2)	72.8 (69.6–75.8)	67.0 (63.0–70.8)	<0.001
Mild	15.4 (14.6–16.3)	12.9 (11.2–14.7)	14.4 (12.9–16.1)	15.7 (14.2–17.4)	16.3 (14.6–18.1)	17.3 (14.9–20.0)	20.2 (17.1–23.6)	
Moderate	4.6 (4.2–5.1)	3.2 (2.5–4.1)	4.0 (3.1–5.1)	4.3 (3.6–5.1)	4.9 (4.1–5.9)	6.4 (5.0–8.1)	9.3 (6.9–12.4)	
Severe	2.3 (2.0–2.6)	1.0 (0.7–1.4)	2.2 (1.7–2.8)	2.4 (1.8–3.2)	2.5 (2.0–3.2)	3.5 (2.6–4.7)	3.5 (2.4–5.1)	
**Antidepressants use**	12.6 (11.8–13.4)	7.5 (6.1–9.2)	9.6 (8.1–11.2)	11.6 (10.1–13.2)	15.5 (14.0–17.1)	18.4 (15.9–21.2)	23.9 (19.9–28.3)	<0.001

Abbreviations: BMI, body mass index; MetS, metabolic syndrome. ^a^ Prevalence estimates [% (95%CI)] are weighted to be nationally representative.

**Table 3:** Odds ratios for individual and clustered MetS components by depressive symptoms ^a^.

	**Individual MetS Components**				
**Depressive symptoms**	Hypertension	Raised triglyceride	Reduced HDL-C	Central obesity	Raised blood glucose
Normal	1 (ref)	1 (ref)	1 (ref)	1 (ref)	1 (ref)
Mild	1.12 (0.97–1.29)	**1.30 (1.12–1.52)**	1.05 (0.90–1.24)	1.08 (0.89–1.32)	1.12 (0.95–1.32)
Moderate	**1.37 (1.09–1.72)**	**1.63 (1.25–2.14)**	1.22 (1.00–1.49)	**1.82 (1.21–2.74)**	**1.37 (1.05–1.79)**
Severe	1.23 (0.82–1.84)	**1.44 (1.05–1.97)**	1.18 (0.87–1.59)	1.00 (0.63–1.61)	1.36 (0.98–1.90)
	**Clustered Number of MetS Components**				
**Depressive symptoms**	One	Two	Three	Four	Five
Normal	1 (ref)	1 (ref)	1 (ref)	1 (ref)	1 (ref)
Mild	1.15 (0.94–1.41)	1.23 (0.96–1.58)	1.25 (0.96–1.62)	1.34 (0.99–1.83)	**1.82 (1.25–2.67)**
Moderate	1.52 (1.00–2.31)	**1.69 (1.09–2.62)**	**1.90 (1.24–2.91)**	**2.63 (1.52–4.55)**	**4.31 (2.45–7.60)**
Severe	**2.08 (1.29–3.37)**	**2.11 (1.24–3.62)**	**2.21 (1.18–4.12)**	**3.04 (1.68–5.51)**	**3.35 (1.57–7.14)**

Abbreviations: HDL-C, high-density lipoprotein-cholesterol; MetS, metabolic syndrome. Model was adjusted for: age, sex, race, education, family income-to-poverty ratio, insurance, marital status, physical activity, BMI, smoking status, and alcohol consumption. ^a^ The values of odds ratio (95%CI) with boldface indicates statistical significance (*p* < 0.05).

**Table 4:** Odds ratios for individual and clustered MetS components by antidepressant use ^a^.

	**Individual MetS Components**				
	Hypertension	Raised triglyceride	Reduced HDL-C	Central obesity	Raised blood glucose
**Antidepressant use**	**1.40 (1.14–1.72)**	**1.43 (1.17–1.74)**	1.01 (0.84–1.22)	1.14 (0.88–1.48)	1.03 (0.88–1.21)
	**Clustered Number of MetS Components**				
	One	Two	Three	Four	Five
**Antidepressant use**	1.07 (0.78–1.47)	1.04 (0.75–1.44)	1.32 (0.88–1.97)	1.44 (0.99–2.09)	**1.74 (1.13–2.68)**

Abbreviations: HDL-C, high-density lipoprotein-cholesterol; MetS, metabolic syndrome. Model was adjusted for: age, sex, race, education, family income-to-poverty ratio, insurance, marital status, physical activity, BMI, smoking status, alcohol consumption, and depressive symptoms. ^a^ The value of odds ratio (95%CI) with boldface indicates statistical significance (*p* < 0.05).

## Data Availability

Data used in this study are available from the published NHANES database (https://www.cdc.gov/nchs/nhanes/index.htm (accessed on 2 October 2022)).

## Supplementary Materials

The following supporting information can be downloaded at: https://www.mdpi.com/article/10.3390/jcm12123891/s1, Figure S1: Prevalence of individual (A) and clustered (B) MetS components by depressive symptoms in men and women; Figure S2: Prevalence of individual (A) and clustered (B) MetS components by antidepression use in men and women; Table S1: Characteristics of participants by individual MetS components; Table S2: Characteristics of participants by sex; Table S3: Depressive symptoms and antidepressant use by clustered and individual MetS components in men and women; Table S4: Odds ratios for individual and clustered MetS components by depressive symptoms in men and women; Table S5: Beta values and *p* values for models of depressive symptoms; Table S6: Odds ratios for individual and clustered MetS components by antidepressant use; Table S7: Beta values and *p* values for models of antidepressant use; Table S8: Odds ratios for individual and clustered MetS components by depressive symptoms in men and women; Table S9: Odds ratios for individual and clustered MetS components by antidepressant use in men and women.
