# Supplementary material for: Dissecting Relations between Depression Severity, Antidepressant Use, and Metabolic Syndrome Components in the NHANES 2005–2020
Source: J Clin Med. 2023 Jun 7;12(12):3891. doi: 10.3390/jcm12123891 (PMC10299566; doi:10.3390/jcm12123891)
Supplement: Supplementary file 1 [file jcm-12-03891-s001.zip › jcm-2323653-supplementary.pdf]

Supplementary Information

Table S1. Characteristics of participants by individual MetS components.

Table S2. Characteristics of participants by sex.

Table S3. Depressive symptoms and antidepressant use by clustered and individual MetS components in men and women.

Table S4. Odds ratios for individual and clustered MetS components by depressive symptoms.

Table S5. Beta values and *p* values for models of depressive symptoms.

Table S6. Odds ratios for individual and clustered MetS components by antidepressant use.

Table S7. Beta values and *p* values for models of antidepressant use.

Table S8. Odds ratios for individual and clustered MetS components by depressive symptoms in men and women.

Table S9. Odds ratios for individual and clustered MetS components by antidepressant use in men and women.

Figure S1. Prevalence of individual (A) and clustered (B) MetS components by depressive symptoms in men and women.

Figure S2. Prevalence of individual (A) and clustered (B) MetS components by antidepression use in men and women.

Table S1. Characteristics of participants by individual MetS components.[a]

**Characteristic**	**Total (N=15315)**	**Hypertension**			**Raised triglyceride**			**Reduced HDL-C**			**Central obesity**			**Raised blood glucose**		
		**No (*n*=8363, 60.0 [58.7-61.3])**	**Yes (*n*=6952, 40.0 [38.7-41.3])**	***P* value**	**No (*n*=11532, 75.6 [74.6-76.6])**	**Yes (*n*=3783, 24.4 [23.4-25.4])**	***P* value**	**No (*n*=11056, 73.0 [71.8-74.2])**	**Yes (*n*=4259, 27.0 [25.8-28.2])**	***P* value**	**No (*n*=6535, 43.7 [42.3-45.1])**	**Yes (*n*=8780, 56.3 [54.9-57.7])**	***P* value**	**No (*n*=6822, 48.1 [46.6-49.5])**	**Yes (*n*=8493, 51.9 [50.5-53.4])**	***P* value**
**Age, years**																
20-44	45.5 (44.0-47.0)	62.3 (60.5-64.2)	20.1 (18.8-21.6)	<0.001	47.6 (45.9-49.2)	39.1 (36.6-41.6)	<0.001	44.1 (42.4-45.9)	49.2 (47.1-51.2)	<0.001	55.7 (53.7-57.7)	37.5 (35.9-39.2)	<0.001	60.2 (58.3-62.1)	31.9 (30.1-33.7)	<0.001
45-64	36.4 (35.2-37.6)	29.9 (28.3-31.5)	46.1 (44.6-47.7)		34.6 (33.3-36.0)	41.7 (39.3-44.1)		36.6 (35.2-38.0)	35.7 (33.8-37.6)		31.8 (29.9-33.6)	39.9 (38.6-41.3)		29.5 (27.9-31.2)	42.7 (41.1-44.3)	
≥65	18.2 (17.2-19.2)	7.8 (7.0-8.6)	33.7 (32.1-35.4)		17.8 (16.7-19.0)	19.2 (17.7-20.9)		19.3 (18.1-20.4)	15.1 (13.7-16.7)		12.5 (11.4-13.7)	22.5 (21.3-23.8)		10.3 (9.3-11.4)	25.5 (24.1-26.8)	
**Sex**																
Men	49.4 (48.4-50.3)	47.8 (46.5-49.1)	51.7 (50.3-53.1)	<0.001	46.9 (45.7-48.1)	57.0 (55.0-59.1)	<0.001	50.8 (49.6-52.0)	45.5 (43.5-47.4)	<0.001	61.5 (59.8-63.2)	40.0 (38.5-41.4)	<0.001	41.2 (39.7-42.6)	57.0 (55.5-58.4)	<0.001
Women	50.6 (49.7-51.6)	52.2 (50.9-53.5)	48.3 (46.9-49.7)		53.1 (51.9-54.3)	43.0 (40.9-45.0)		49.2 (48.0-50.4)	54.5 (52.6-56.5)		38.5 (36.8-40.2)	60.0 (58.6-61.5)		58.8 (57.4-60.3)	43.0 (41.6-44.5)	
**Race/ethnicity**																
Non-Hispanic White	67.8 (65.4-70.1)	66.9 (64.5-69.2)	69.1 (66.2-71.9)		66.6 (64.1-69.0)	71.5 (68.8-74.0)		68.2 (65.7-70.5)	66.7 (63.7-69.6)		65.2 (62.7-67.6)	69.8 (67.1-72.4)		67.8 (65.0-70.4)	67.8 (65.3-70.3)	
Non-Hispanic Black	10.8 (9.6-12.2)	8.7 (7.7-9.9)	14.0 (12.2-16.0)	<0.001	12.6 (11.2-14.2)	5.2 (4.4-6.2)	<0.001	11.5 (10.1-13.0)	9.1 (7.8-10.6)	<0.001	10.1 (9.0-11.4)	11.3 (9.9-13.0)	<0.001	12.0 (10.5-13.6)	9.8 (8.6-11.1)	<0.001
Mexican American	8.4 (7.2-9.7)	10.1 (8.7-11.7)	5.8 (4.8-7)		7.8 (6.7-9.1)	10.2 (8.7-11.9)		7.8 (6.7-9)	10.0 (8.5-11.7)		8.3 (7.2-9.6)	8.4 (7.1-10.0)		7.6 (6.5-8.8)	9.1 (7.8-10.7)	
Other Hispanic	5.7 (4.8-6.6)	6.4 (5.4-7.5)	4.6 (3.8-5.6)		5.7 (4.9-6.6)	5.7 (4.5-7.1)		5.2 (4.4-6.1)	6.9 (5.8-8.2)		6.1 (5.1-7.3)	5.3 (4.5-6.3)		5.7 (4.8-6.8)	5.7 (4.8-6.7)	
Other race	7.3 (6.6-8.2)	7.9 (7.1-8.8)	6.5 (5.6-7.5)		7.3 (6.5-8.2)	7.4 (6.4-8.6)		7.4 (6.6-8.2)	7.3 (6.2-8.6)		10.2 (9.1-11.5)	5.1 (4.4-5.9)		7.0 (6.2-8)	7.6 (6.7-8.6)	
**Education**																
<High school	15.1 (14-16.3)	13.6 (12.3-15.0)	17.3 (16.0-18.7)		14.1 (13.0-15.3)	18.3 (16.7-20.0)		13.7 (12.5-15.0)	18.9 (17.3-20.6)		14.3 (13.0-15.7)	15.7 (14.5-17.0)		12.6 (11.2-14.1)	17.4 (16.1-18.8)	
High school	23.5 (22.3-24.8)	21.1 (19.7-22.7)	27.0 (25.4-28.8)	<0.001	23.0 (21.7-24.3)	25.1 (22.9-27.5)	<0.001	23.0 (21.7-24.4)	24.7 (22.6-26.9)	<0.001	22.0 (20.3-23.8)	24.6 (23.3-26.0)	<0.001	21.2 (19.7-22.8)	25.6 (24.1-27.2)	<0.001
Some college	31.0 (29.8-32.3)	31.1 (29.6-32.6)	31.0 (29.1-32.9)		30.7 (29.3-32.1)	32.1 (30.0-34.3)		30.3 (29.0-31.7)	33.0 (30.8-35.2)		28.1 (26.4-29.8)	33.3 (31.8-34.9)		32.6 (30.9-34.3)	29.6 (28.0-31.4)	
College or higher	30.4 (28.4-32.4)	34.2 (31.9-36.5)	24.7 (22.6-26.9)		32.3 (30.2-34.4)	24.5 (22.1-27.1)		32.9 (30.9-35.0)	23.4 (21.2-25.9)		35.6 (33.2-38.0)	26.3 (24.3-28.5)		33.7 (31.4-36.0)	27.3 (25.3-29.5)	
**Family income-to-poverty ratio**																
<130%	20.0 (18.6-21.4)	20.4 (19.0-21.9)	19.3 (17.7-21.1)	0.05	19.6 (18.1-21.1)	21.2 (19.4-23.2)	0.09	18.0 (16.7-19.5)	25.2 (23.3-27.2)	<0.001	19.4 (17.8-21.0)	20.4 (18.9-22.0)	<0.001	20.1 (18.5-21.8)	19.8 (18.4-21.3)	0.16
130%-349%	36.1 (34.8-37.5)	35.1 (33.5-36.7)	37.7 (35.9-39.5)		35.9 (34.4-37.5)	36.8 (34.7-38.9)		35.2 (33.7-36.8)	38.5 (36.4-40.7)		33.8 (32.1-35.5)	38.0 (36.1-39.8)		35.0 (33.2-37.0)	37.2 (35.5-38.9)	
≥350%	43.9 (42.0-45.9)	44.5 (42.3-46.7)	42.9 (40.6-45.4)		44.5 (42.4-46.6)	42.0 (39.6-44.5)		46.7 (44.8-48.6)	36.3 (33.3-39.4)		46.8 (44.5-49.2)	41.6 (39.3-44.0)		44.9 (42.3-47.4)	43.0 (40.8-45.3)	

**Health insurance**																
No	17.1 (16.0-18.3)	20.5 (19.0-22.2)	12.0 (10.9-13.2)	<0.001	17.0 (15.8-18.2)	17.5 (15.7-19.5)	0.54	16.1 (14.8-17.4)	20.0 (18.3-21.7)	<0.001	20.7 (19.1-22.5)	14.3 (13.1-15.6)	<0.001	19.2 (17.7-20.9)	15.2 (14.0-16.4)	<0.001
Yes	82.9 (81.7-84.0)	79.5 (77.8-81.0)	88.0 (86.8-89.1)		83.0 (81.8-84.2)	82.5 (80.5-84.3)		83.9 (82.6-85.2)	80.0 (78.3-81.7)		79.3 (77.5-80.9)	85.7 (84.4-86.9)		80.8 (79.1-82.3)	84.8 (83.6-86.0)	
**Marital status**																
Married/Living with Partner	64.3 (62.6-65.9)	63.6 (61.7-65.4)	65.3 (63.3-67.2)		63.3 (61.5-65.0)	67.3 (64.7-69.9)		64.2 (62.3-66.0)	64.5 (62.0-67.0)		62.4 (60.3-64.5)	65.7 (63.6-67.8)		61.6 (59.7-63.5)	66.7 (64.7-68.7)	
Widowed/Divorced/Separated	17.7 (16.7-18.7)	13.3 (12.3-14.3)	24.3 (22.7-25.9)	<0.001	17.2 (16.2-18.3)	19.1 (17.4-21.0)	<0.001	17.5 (16.4-18.7)	18.1 (16.6-19.7)	0.54	13.9 (12.7-15.2)	20.6 (19.2-22)	<0.001	14.5 (13.4-15.6)	20.7 (19.2-22.2)	<0.001
Never married	18.1 (16.8-19.4)	23.1 (21.4-24.9)	10.5 (9.5-11.6)		19.5 (18.1-21.0)	13.5 (11.8-15.4)		18.3 (17.0-19.7)	17.3 (15.4-19.4)		23.7 (21.8-25.7)	13.7 (12.4-15)		23.9 (22.2-25.7)	12.6 (11.3-14.1)	
**BMI, kg/m^2**																
Normal (<25)	29.6 (28.5-30.7)	36.8 (35.2-38.4)	18.7 (17.6-19.9)		34.9 (33.6-36.2)	13.0 (11.6-14.4)		35.4 (34.0-36.8)	13.7 (12.3-15.3)		61.4 (60.2-62.7)	4.8 (4.1-5.6)		41.6 (39.8-43.4)	18.4 (17.3-19.6)	
Overweight (25-<30)	33.2 (32.4-34.1)	33.8 (32.7-35.0)	32.3 (30.9-33.8)	<0.001	32.5 (31.5-33.6)	35.4 (33.6-37.2)	<0.001	34.2 (33.1-35.3)	30.6 (28.7-32.6)	<0.001	35.7 (34.5-37.0)	31.3 (29.9-32.7)	<0.001	32.4 (31.1-33.7)	34.0 (32.8-35.3)	<0.001
Obese (≥30)	37.2 (36.1-38.4)	29.4 (27.9-31.0)	49 (47.3-50.6)		32.6 (31.3-33.9)	51.6 (49.5-53.7)		30.4 (29.1-31.7)	55.7 (53.4-58.0)		2.8 (2.4-3.4)	63.9 (62.4-65.4)		26 (24.5-27.6)	47.6 (46.1-49.1)	
**Physical activity**																
Little/None	49.9 (48.5-51.3)	48.1 (46.4-49.9)	52.5 (50.7-54.3)	<0.001	50.3 (48.8-51.9)	48.3 (46.0-50.7)	0.04	49.3 (47.8-50.9)	51.3 (49.3-53.4)	0.17	47.6 (45.7-49.5)	51.6 (50.1-53.2)	<0.001	49.1 (47.1-51.1)	50.6 (48.9-52.3)	0.17
Moderate	25.3 (24.2-26.4)	24.7 (23.4-26.0)	26.1 (24.5-27.7)		24.6 (23.5-25.7)	27.3 (25.2-29.6)		25.4 (24.2-26.5)	25.0 (23.1-27.1)		23.8 (22.3-25.3)	26.4 (25.1-27.7)		25.1 (23.7-26.6)	25.4 (24.0-26.9)	
Vigorous	24.9 (23.8-26.0)	27.2 (25.7-28.7)	21.4 (20.0-22.9)		25.1 (23.8-26.3)	24.3 (22.5-26.3)		25.3 (24.1-26.6)	23.6 (21.9-25.5)		28.6 (26.9-30.4)	22.0 (20.7-23.2)		25.8 (24.4-27.3)	24.0 (22.7-25.4)	
**Smoking status**																
Former/Never	80.3 (79.2-81.5)	78.8 (77.2-80.3)	82.7 (81.3-84)	<0.001	81.3 (80-82.6)	77.3 (75.5-79)	<0.001	82.2 (81-83.4)	75.2 (73.2-77.1)	<0.001	77.1 (75.4-78.8)	82.8 (81.5-84)	<0.001	79.0 (77.4-80.6)	81.6 (80.2-82.8)	0.01
Current	19.7 (18.5-20.8)	21.2 (19.7-22.8)	17.3 (16-18.7)		18.7 (17.4-20)	22.7 (21-24.5)		17.8 (16.6-19)	24.8 (22.9-26.8)		22.9 (21.2-24.6)	17.2 (16-18.5)		21.0 (19.4-22.6)	18.4 (17.2-19.8)	
**Alcohol consumption**																
Non-drinker	21.3 (20.1-22.6)	17.7 (16.4-19.1)	26.8 (25.2-28.5)	<0.001	20.6 (19.3-22.0)	23.5 (21.7-25.4)	<0.001	19.4 (18.2-20.7)	26.5 (24.6-28.5)	<0.001	16.8 (15.5-18.3)	24.8 (23.3-26.4)	<0.001	19.2 (17.7-20.8)	23.3 (21.8-24.8)	<0.001
Moderate drinker	62.8 (61.1-64.4)	65.0 (63.0-66.9)	59.4 (57.2-61.5)		64.4 (62.8-66.0)	57.6 (54.9-60.2)		65.4 (63.8-67.0)	55.5 (53.2-57.9)		66.4 (64.2-68.4)	60.0 (58.1-61.8)		63.9 (61.8-65.9)	61.7 (59.8-63.6)	
Heavy drinker	15.9 (15.0-16.8)	17.3 (16.1-18.6)	13.8 (12.6-15.2)		15 (14.1-15.9)	18.9 (17.0-20.9)		15.1 (14.2-16.1)	18 (16.4-19.7)		16.8 (15.4-18.3)	15.2 (14.2-16.4)		16.9 (15.7-18.1)	15.0 (13.8-16.3)	
**Depressive symptom**																
Minimal	77.6 (76.6-78.6)	78.7 (77.4-79.9)	76.0 (74.5-77.5)	0.01	79.2 (78.1-80.3)	72.7 (70.7-74.7)	<0.001	79.3 (78.2-80.3)	73.1 (71.0-75.2)	<0.001	81.4 (80-82.6)	74.7 (73.3-76.0)	<0.001	78.6 (77.3-79.9)	76.7 (75.2-78.2)	0.08
Mild	15.4 (14.6-16.3)	15.1 (14.1-16.2)	16.0 (14.7-17.3)		14.6 (13.7-15.6)	18.0 (16.4-19.8)		14.7 (13.8-15.7)	17.4 (15.6-19.3)		13.4 (12.2-14.6)	17.1 (15.9-18.3)		15.2 (14.1-16.4)	15.7 (14.5-17.0)	

Moderate	4.6 (4.2-5.1)	4.1 (3.6-4.7)	5.4 (4.7-6.2)		4.2 (3.7-4.7)	6.1 (5.2-7.3)		4.1 (3.7-4.6)	6.1 (5.3-7.1)		3.5 (3.0-4.1)	5.5 (4.9-6.2)		4.2 (3.6-4.9)	5.0 (4.4-5.7)	
Severe	2.3 (2.0-2.6)	2.1 (1.7-2.5)	2.6 (2.1-3.2)		2.0 (1.8-2.4)	3.1 (2.5-3.8)		1.9 (1.6-2.2)	3.4 (2.7-4.1)		1.7 (1.4-2.2)	2.7 (2.4-3.2)		2.0 (1.7-2.5)	2.6 (2.2-3.0)	
**Antidepressants use**																
No	87.4 (86.6-88.2)	90.1 (88.9-91.1)	83.5 (82.2-84.6)	<0.001	88.9 (88.1-89.7)	82.8 (80.7-84.7)	<0.001	88.2 (87.2-89.1)	85.4 (83.7-87.0)	0.003	92.2 (91.2-93.0)	83.7 (82.6-84.8)	<0.001	88.7 (87.5-89.8)	86.3 (85.2-87.2)	<0.001
Yes	12.6 (11.8-13.4)	9.9 (8.9-11.1)	16.5 (15.4-17.8)		11.1 (10.3-11.9)	17.2 (15.3-19.3)		11.8 (10.9-12.8)	14.6 (13.0-16.3)		7.8 (7.0-8.8)	16.3 (15.2-17.4)		11.3 (10.2-12.5)	13.7 (12.8-14.8)	

Abbreviations: BMI, body mass index; HDL-C, high-density lipoprotein-cholesterol; MetS, metabolic syndrome.

[a] Prevalence estimates [% (95% CI)] are weighted to be nationally representative.

Table S2. Characteristics of participants by gender. [a]

Characteristic	Total (N=15315)		Men (n=7575)		Women (n=7740)		P value
	Unweighted No.	% (95%CI)	Unweighted No.	% (95%CI)	Unweighted No.	% (95%CI)	
**Age, years**							
20-44	6326	45.5 (44.0-47.0)	3096	47.3 (45.4-49.1)	3230	43.8 (42.1-45.4)	
45-64	5424	36.4 (35.2-37.6)	2664	36.0 (34.5-37.5)	2760	36.7 (35.3-38.1)	<0.001
≥65	3565	18.2 (17.2-19.2)	1815	16.8 (15.7-17.9)	1750	19.5 (18.3-20.8)	
**Race/ethnicity**							
Non-Hispanic White	6590	67.8 (65.4-70.1)	3331	67.8 (65.4-70.2)	3259	67.7 (65.1-70.2)	
Non-Hispanic Black	3122	10.8 (9.6-12.2)	1498	10.0 (8.9-11.3)	1624	11.6 (10.2-13.2)	
Mexican American	2381	8.4 (7.2-9.7)	1175	9.1 (7.9-10.5)	1206	7.7 (6.5-9.0)	<0.001
Other Hispanic	1567	5.7 (4.8-6.6)	744	5.7 (4.8-6.8)	823	5.6 (4.8-6.6)	
Other race	1655	7.3 (6.6-8.2)	827	7.3 (6.4-8.2)	828	7.4 (6.6-8.4)	
**Education**							
<High school	3543	15.1 (14.0-16.3)	1839	16.2 (14.8-17.8)	1704	14.0 (12.9-15.1)	
High school	3534	23.5 (22.3-24.8)	1819	24.0 (22.3-25.8)	1715	22.9 (21.7-24.3)	<0.001
Some college	4557	31.0 (29.8-32.3)	2080	29.4 (27.8-30.9)	2477	32.7 (31.2-34.2)	
College or higher	3673	30.4 (28.4-32.4)	1833	30.4 (28.2-32.6)	1840	30.4 (28.4-32.4)	
**Family income-to-poverty ratio**							
<130%	4168	20.0 (18.6-21.4)	1920	18.4 (17.0-19.8)	2248	21.5 (20.0-23.1)	
130%-349%	5375	36.1 (34.8-37.5)	2663	35.4 (33.8-37.0)	2712	36.9 (35.2-38.6)	<0.001
≥350%	4488	43.9 (42.0-45.9)	2355	46.3 (44.2-48.4)	2133	41.6 (39.4-43.8)	
**Health insurance**							
No	3214	17.1 (16.0-18.3)	1785	19.9 (18.5-21.4)	1429	14.4 (13.2-15.6)	<0.001
Yes	12084	82.9 (81.7-84)	5783	80.1 (78.6-81.5)	6301	85.6 (84.4-86.8)	
**Marital status**							
Married/Living with Partner	9337	64.3 (62.6-65.9)	5027	67.3 (65.5-69.1)	4310	61.3 (59.3-63.2)	
Widowed/Divorced/Separated	3250	17.7 (16.7-18.7)	1181	12.8 (11.8-13.9)	2069	22.4 (21.1-23.8)	<0.001
Never married	2724	18.1 (16.8-19.4)	1366	19.8 (18.3-21.5)	1358	16.3 (14.9-17.8)	
**BMI, kg/m²**							
Normal (<25)	4300	29.6 (28.5-30.7)	2016	26.2 (24.7-27.7)	2284	32.9 (31.4-34.3)	<0.001

Overweight (25-<30)	5103	33.2 (32.4-34.1)	2908	38.7 (37.4-40.0)	2195	27.9 (26.6-29.3)	
Obese (≥30)	5875	37.2 (36.1-38.4)	2622	35.1 (33.3-37.0)	3253	39.2 (37.7-40.7)	
**Physical activity**							
Little/None	8354	49.9 (48.5-51.3)	3674	43.6 (41.7-45.5)	4680	56.0 (54.2-57.7)	
Moderate	3531	25.3 (24.2-26.4)	1644	23.4 (22-24.9)	1887	27.1 (25.7-28.4)	<0.001
Vigorous	3430	24.9 (23.8-26.0)	2257	33.0 (31.4-34.7)	1173	16.9 (15.7-18.3)	
**Smoking status**							
Former/Never	12252	80.3 (79.2-81.5)	5799	77.9 (76.4-79.4)	6453	82.7 (81.4-83.9)	<0.001
Current	3052	19.7 (18.5-20.8)	1771	22.1 (20.6-23.6)	1281	17.3 (16.1-18.6)	
**Alcohol consumption**							
Non-drinker	4143	21.3 (20.1-22.6)	1404	14.7 (13.5-16.0)	2739	27.8 (26.2-29.4)	
Moderate drinker	8663	62.8 (61.1-64.4)	4457	63.8 (61.9-65.8)	4206	61.7 (59.8-63.6)	<0.001
Heavy drinker	2492	15.9 (15.0-16.8)	1704	21.5 (20.0-23.0)	788	10.5 (9.6-11.5)	
**Depressive symptom**							
Normal	11682	77.6 (76.6-78.6)	6180	82.1 (81.0-83.2)	5502	73.2 (71.8-74.7)	
Mild	2373	15.4 (14.6-16.3)	947	12.8 (11.8-13.9)	1426	18.0 (16.8-19.2)	<0.001
Moderate	823	4.6 (4.2-5.1)	294	3.3 (2.8-3.8)	529	6.0 (5.3-6.7)	
Severe	437	2.3 (2.0-2.6)	154	1.8 (1.5-2.2)	283	2.8 (2.4-3.3)	
**Antidepressants use**							
No	13738	87.4 (86.6-88.2)	7085	92.6 (91.8-93.3)	6653	82.4 (81.1-83.6)	<0.001
Yes	1577	12.6 (11.8-13.4)	490	7.4 (6.7-8.2)	1087	17.6 (16.4-18.9)	
**Clustering MetS components**							
None	2230	17.1 (16.1-18.2)	1051	16.1 (14.7-17.5)	1179	18.2 (16.8-19.6)	
One	3322	23.3 (22.3-24.3)	1732	24.1 (22.7-25.6)	1590	22.5 (21.1-23.8)	
Two	3701	23.2 (22.2-24.2)	1854	23.2 (21.8-24.6)	1847	23.2 (21.9-24.5)	0.08
Three	3483	20.7 (19.8-21.6)	1747	21.3 (19.9-22.6)	1736	20.1 (19-21.3)	
Four	1801	10.8 (10.1-11.6)	842	10.8 (9.7-12.1)	959	10.8 (9.8-11.8)	
Five	778	4.9 (4.5-5.4)	349	4.6 (4.0-5.2)	429	5.3 (4.7-6)	
**MetS status**							
No	9253	63.6 (62.3-64.9)	4637	63.4 (61.5-65.2)	4616	63.8 (62.1-65.4)	0.72
Yes	6062	36.4 (35.1-37.7)	2938	36.6 (34.8-38.5)	3124	36.2 (34.6-37.9)	
**Hypertension**							
No	8363	60 (58.7-61.3)	3937	58.1 (56.5-59.8)	4426	61.9 (60.3-63.4)	<0.001
Yes	6952	40 (38.7-41.3)	3638	41.9 (40.2-43.5)	3314	38.1 (36.6-39.7)	
**Raised triglyceride**							

No	11532	75.6 (74.6-76.6)	5473	71.8 (70.3-73.3)	6059	79.3 (78-80.5)	<0.001
Yes	3783	24.4 (23.4-25.4)	2102	28.2 (26.7-29.7)	1681	20.7 (19.5-22)	
**Reduced HDL-C**							
No	11056	73 (71.8-74.2)	5706	75.2 (73.6-76.7)	5350	70.9 (69.4-72.4)	<0.001
Yes	4259	27 (25.8-28.2)	1869	24.8 (23.3-26.4)	2390	29.1 (27.6-30.6)	
**Central obesity**							
No	6535	43.7 (42.3-45.1)	4180	54.5 (52.4-56.5)	2355	33.2 (31.7-34.8)	<0.001
Yes	8780	56.3 (54.9-57.7)	3395	45.5 (43.5-47.6)	5385	66.8 (65.2-68.3)	
**Elevated blood glucose**							
No	6822	48.1 (46.6-49.5)	2785	40.1 (38.2-42.1)	4037	55.9 (54.2-57.5)	<0.001
Yes	8493	51.9 (50.5-53.4)	4790	59.9 (57.9-61.8)	3703	44.1 (42.5-45.8)	

Abbreviations: BMI, body mass index; CI, confidence intervals; HDL-C, high-density lipoprotein-cholesterol; MetS, metabolic syndrome.

[a] Prevalence estimates [% (95% CI)] are weighted to be nationally representative.

Table S3. Depressive symptoms and antidepressant use by individual and clustered MetS components in men and women.[a]

	Individual MetS components									
	Hypertension (*n*=6952)		Raised triglyceride (*n*=3783)		Reduced HDL-C (*n*=4259)		Central obesity (*n*=8780)		Elevated blood glucose (*n*=8493)	
	**% (95%CI)**	***P* value [b]**	**% (95%CI)**	***P* value [b]**	**% (95%CI)**	***P* value [b]**	**% (95%CI)**	***P* value [b]**	**% (95%CI)**	***P* value [b]**
**Men**										
Depressive symptoms										
Normal	82 (80.2-83.6)	0.56	78.4 (75.8-80.8)	<0.001	78.9 (75.9-81.5)	0.02	81.6 (79.8-83.3)	0.85	82.3 (80.7-83.8)	0.95
Mild	12.6 (11.1-14.2)		14.8 (12.7-17.1)		14.9 (12.6-17.5)		13.3 (11.7-15)		12.8 (11.3-14.4)	
Moderate	3.3 (2.7-4.1)		4.1 (3.1-5.5)		3.9 (3.0-5.2)		3.3 (2.7-4.1)		3.2 (2.7-3.8)	
Severe	2.1 (1.5-2.9)		2.7 (2.0-3.7)		2.3 (1.6-3.3)		1.8 (1.4-2.3)		1.8 (1.4-2.3)	
Antidepressant use										
No	89.8 (88.3-91.1)	<0.001	88.5 (86.2-90.4)	<0.001	90.6 (88.6-92.3)	0.01	90.6 (89.2-91.8)	<0.001	91.6 (90.4-92.7)	0.01
Yes	10.2 (8.9-11.7)		11.5 (9.6-13.8)		9.4 (7.7-11.4)		9.4 (8.2-10.8)		8.4 (7.3-9.6)	
**Women**										
Depressive symptoms										
Normal	69.6 (67.1-72.1)	<0.001	65.2 (62.0-68.3)	<0.001	68.4 (65.8-70.9)	<0.001	70.1 (68.5-71.7)	<0.001	69.3 (67-71.6)	<0.001
Mild	19.6 (17.6-21.8)		22.4 (19.6-25.4)		19.5 (17.3-21.8)		19.6 (18.2-21)		19.6 (17.8-21.4)	
Moderate	7.6 (6.5-8.9)		8.8 (7.1-10.9)		7.9 (6.6-9.5)		6.9 (6.1-7.9)		7.5 (6.4-8.8)	
Severe	3.1 (2.5-3.9)		3.6 (2.8-4.7)		4.2 (3.4-5.2)		3.4 (2.8-4)		3.6 (2.9-4.4)	
Antidepressant use										
No	76.7 (74.9-78.4)	<0.001	75.2 (71.9-78.2)	<0.001	81 (78.6-83.2)	0.18	79.2 (77.6-80.7)	<0.001	79.2 (77.2-81)	<0.001
Yes	23.3 (21.6-25.1)		24.8 (21.8-28.1)		19 (16.8-21.4)		20.8 (19.3-22.4)		20.8 (19.0-22.8)	

	Clustered number of MetS components					
	**One (*n*=3322)**	**Two (*n*=3701)**	**Three (*n*=3483)**	**Four (*n*=1801)**	**Five (*n*=778)**	***P* value**
**Men**						
Depressive symptoms						
Normal	82.3 (79.9-84.5)	80.8 (78-83.3)	83.6 (81.2-85.8)	80.8 (77.0-84.1)	73.9 (66.9-79.8)	0.04
Mild	12.3 (10.5-14.4)	13.9 (11.4-16.7)	11.8 (9.8-14.2)	14.2 (11.4-17.5)	16.2 (11.6-22.2)	
Moderate	3.3 (2.3-4.6)	3.6 (2.7-4.7)	2.9 (2.2-3.8)	2.4 (1.5-3.9)	7.0 (4.5-11)	
Severe	2.1 (1.4-3.1)	1.8 (1.1-2.8)	1.6 (1-2.6)	2.5 (1.4-4.4)	2.8 (1.4-5.9)	
Antidepressant use						
No	95.5 (94.1-96.6)	93.9 (92.0-95.3)	89.6 (87.6-91.2)	89.3 (85.6-92.1)	82.7 (76.4-87.6)	<0.001

Yes	4.5 (3.4-5.9)	6.1 (4.7-8)	10.4 (8.8-12.4)	10.7 (7.9-14.4)	17.3 (12.4-23.6)	
**Women**						
Depressive symptoms						
Normal	76.3 (72.8-79.5)	74.5 (71.9-76.9)	68.7 (65.5-71.8)	64.9 (59.9-69.6)	61.2 (56.3-65.9)	
Mild	16.6 (14.3-19.3)	17.5 (15.5-19.7)	20.8 (18.2-23.7)	20.4 (16.6-24.8)	23.5 (19.1-28.5)	<0.001
Moderate	4.7 (3.3-6.8)	5.0 (4.0-6.3)	7.0 (5.6-8.7)	10.3 (7.9-13.1)	11.2 (7.9-15.7)	
Severe	2.3 (1.5-3.4)	3.0 (2.1-4.2)	3.4 (2.7-4.4)	4.5 (3.2-6.2)	4.1 (2.5-6.6)	
Antidepressant use						
No	85.1 (82.2-87.6)	83.2 (80.6-85.5)	79.3 (76.8-81.7)	74.0 (70.4-77.3)	70.6 (64.4-76.2)	<0.001
Yes	14.9 (12.4-17.8)	16.8 (14.5-19.4)	20.7 (18.3-23.2)	26.0 (22.7-29.6)	29.4 (23.8-35.6)	

Abbreviations: HDL-C, high-density lipoprotein-cholesterol; MetS, metabolic syndrome.

[a] Prevalence estimates [% (95% CI)] are weighted to be nationally representative.

[b] *P* value for each abnormity of MetS component versus its corresponding normal group.

Table S4. Odds ratios for individual and clustered MetS components by depressive symptoms.[a]

Depressive symptoms	Individual MetS components									
	Hypertension		Raised triglyceride		Reduced HDL-C		Central obesity		Raised blood glucose	
	Model 1	Model 2	Model 1	Model 2	Model 1	Model 2	Model 1	Model 2	Model 1	Model 2
Normal	1 (ref)	1 (ref)	1 (ref)	1 (ref)	1 (ref)	1 (ref)	1 (ref)	1 (ref)	1 (ref)	1 (ref)
Mild	1.12 (0.97-1.29)	1.07 (0.92-1.24)	**1.30 (1.12-1.52)**	**1.24 (1.06-1.45)**	1.05 (0.90-1.24)	1.05 (0.90-1.23)	1.08 (0.89-1.32)	1.06 (0.87-1.29)	1.12 (0.95-1.32)	1.11 (0.94-1.32)
Moderate	**1.37 (1.09-1.72)**	1.25 (0.97-1.60)	**1.63 (1.25-2.14)**	**1.48 (1.12-1.95)**	1.22 (1.00-1.49)	1.21 (0.99-1.49)	**1.82 (1.21-2.74)**	**1.77 (1.18-2.67)**	**1.37 (1.05-1.79)**	**1.36 (1.05-1.77)**
Severe	1.23 (0.82-1.84)	1.11 (0.74-1.66)	**1.44 (1.05-1.97)**	1.29 (0.93-1.78)	1.18 (0.87-1.59)	1.17 (0.87-1.59)	1.00 (0.63-1.61)	0.97 (0.59-1.58)	1.36 (0.98-1.90)	1.35 (0.96-1.90)

Depressive symptoms	Clustered number of MetS components									
	One		Two		Three		Four		Five	
	Model 1	Model 2	Model 1	Model 2	Model 1	Model 2	Model 1	Model 2	Model 1	Model 2
Normal	1 (ref)	1 (ref)	1 (ref)	1 (ref)	1 (ref)	1 (ref)	1 (ref)	1 (ref)	1 (ref)	1 (ref)
Mild	1.15 (0.94-1.41)	1.14 (0.94-1.39)	1.23 (0.96-1.58)	1.22 (0.94-1.59)	1.25 (0.96-1.62)	1.20 (0.92-1.58)	1.34 (0.99-1.83)	1.28 (0.93-1.76)	**1.82 (1.25-2.67)**	**1.67 (1.13-2.47)**
Moderate	1.52 (1.00-2.31)	1.50 (1.00-2.25)	**1.69 (1.09-2.62)**	**1.68 (1.08-2.62)**	**1.90 (1.24-2.91)**	**1.78 (1.15-2.75)**	**2.63 (1.52-4.55)**	**2.40 (1.35-4.28)**	**4.31 (2.45-7.60)**	**3.66 (2.03-6.61)**
Severe	**2.08 (1.29-3.37)**	**2.03 (1.26-3.26)**	**2.11 (1.24-3.62)**	**2.07 (1.23-3.51)**	**2.21 (1.18-4.12)**	**2.02 (1.10-3.72)**	**3.04 (1.68-5.51)**	**2.71 (1.50-4.90)**	**3.35 (1.57-7.14)**	**2.79 (1.31-5.93)**

Abbreviations: HDL-C, high-density lipoprotein-cholesterol; MetS, metabolic syndrome.

Model 1: Adjusted for: age, sex, race, education, family income-to-poverty ratio, insurance, marital status, physical activity, BMI, smoking status, alcohol consumption.

Model 2: Additionally adjusted for antidepressant use.

[a] The values of odds ratio (95%CI) with boldface indicates statistical significance (P<0.05).

Table S5. Beta values and *p* values for models of depressive symptoms.

Individual MetS components																				
	**Hypertension**				**Raised triglyceride**				**Reduced HDL-C**				**Central obesity**				**Raised blood glucose**			
	**Model 1**		**Model 2**		**Model 1**		**Model 2**		**Model 1**		**Model 2**		**Model 1**		**Model 2**		**Model 1**		**Model 2**	
**Depressive symptoms**	Beta value	*p* value	Beta value	*p* value	Beta value	*p* value	Beta value	*p* value	Beta value	*p* value	Beta value	*p* value	Beta value	*p* value	Beta value	*p* value	Beta value	*p* value	Beta value	*p* value
Mild	0.11	0.12	0.07	0.38	0.26	0.001	0.21	0.01	0.05	0.52	0.05	0.53	0.08	0.43	0.06	0.55	0.11	0.19	0.11	0.22
Moderate	0.31	0.01	0.22	0.09	0.49	0.001	0.39	0.01	0.20	0.05	0.19	0.07	0.60	0.001	0.57	0.01	0.32	0.02	0.31	0.02
Severe	0.21	0.31	0.10	0.61	0.36	0.02	0.25	0.13	0.16	0.28	0.16	0.30	0.00	0.99	-0.04	0.89	0.31	0.07	0.30	0.09

Clustered number of MetS components																				
	**One**				**Two**				**Three**				**Four**				**Five**			
	**Model 1**		**Model 2**		**Model 1**		**Model 2**		**Model 1**		**Model 2**		**Model 1**		**Model 2**		**Model 1**		**Model 2**	
**Depressive symptoms**	Beta value	*p* value	Beta value	*p* value	Beta value	*p* value	Beta value	*p* value	Beta value	*p* value	Beta value	*p* value	Beta value	*p* value	Beta value	*p* value	Beta value	*p* value	Beta value	*p* value
Mild	0.14	0.17	0.13	0.19	0.21	0.11	0.20	0.13	0.22	0.10	0.18	0.18	0.30	0.06	0.25	0.13	0.60	<0.001	0.52	0.01
Moderate	0.42	0.05	0.40	0.05	0.52	0.02	0.52	0.02	0.64	<0.001	0.58	0.01	0.97	<0.001	0.88	<0.001	1.46	<0.001	1.30	0.001
Severe	0.73	<0.001	0.71	<0.001	0.75	0.01	0.73	0.01	0.79	0.01	0.70	0.02	1.11	<0.001	0.99	<0.001	1.21	<0.001	1.03	0.01

Abbreviations: HDL-C, high-density lipoprotein-cholesterol; MetS, metabolic syndrome.

Model 1: Adjusted for: age, sex, race, education, family income-to-poverty ratio, insurance, marital status, physical activity, BMI, smoking status, alcohol consumption.

Model 2: Additionally adjusted for antidepressant use.

Table S6. Odds ratios for individual and clustered MetS components by antidepressant use.[a]

	Individual MetS components									
	Hypertension		Raised triglyceride		Reduced HDL-C		Central obesity		Raised blood glucose	
	Model 1	Model 2	Model 1	Model 2	Model 1	Model 2	Model 1	Model 2	Model 1	Model 2
**Antidepressant use**	**1.45 (1.19-1.75)**	**1.40 (1.14-1.72)**	**1.54 (1.27-1.86)**	**1.43 (1.17-1.74)**	1.05 (0.87-1.26)	1.01 (0.84-1.22)	1.19 (0.93-1.53)	1.14 (0.88-1.48)	1.09 (0.94-1.27)	1.03 (0.88-1.21)
	**Clustered number of MetS components**									
	**One**		**Two**		**Three**		**Four**		**Five**	
	Model 1	Model 2	Model 1	Model 2	Model 1	Model 2	Model 1	Model 2	Model 1	Model 2
**Antidepressant use**	1.17 (0.84-1.63)	1.07 (0.78-1.47)	1.17 (0.85-1.60)	1.04 (0.75-1.44)	1.48 (0.99-2.19)	1.32 (0.88-1.97)	**1.69 (1.19-2.42)**	1.44 (0.99-2.09)	**2.23 (1.48-3.37)**	**1.74 (1.13-2.68)**

Abbreviations: HDL-C, high-density lipoprotein-cholesterol; MetS, metabolic syndrome.

Model 1: Adjusted for: age, sex, race, education, family income-to-poverty ratio, insurance, marital status, physical activity, BMI, smoking status, alcohol consumption.

Model 2: Additionally adjusted for depressive symptoms.

[a] The value of odds ratio (95%CI) with boldface indicates statistical significance (P<0.05).

Table S7. Beta values and *p* values for models of antidepressant use.

	Individual MetS components																			
	Hypertension				Raised triglyceride				Reduced HDL-C				Central obesity				Raised blood glucose			
	Model 1		Model 2		Model 1		Model 2		Model 1		Model 2		Model 1		Model 2		Model 1		Model 2	
	Beta value	*p* value	Beta value	*p* value	Beta value	*p* value	Beta value	*p* value	Beta value	*p* value	Beta value	*p* value	Beta value	*p* value	Beta value	*p* value	Beta value	*p* value	Beta value	*p* value
**Antidepressant use**	0.37	<0.001	0.33	0.002	0.43	<0.001	0.35	0.001	0.05	0.61	0.01	0.88	0.18	0.17	0.13	0.32	0.09	0.25	0.03	0.7

	Clustered number of MetS components																			
	One				Two				Three				Four				Five			
	Model 1		Model 2		Model 1		Model 2		Model 1		Model 2		Model 1		Model 2		Model 1		Model 2	
	Beta value	*p* value	Beta value	*p* value	Beta value	*p* value	Beta value	*p* value	Beta value	*p* value	Beta value	*p* value	Beta value	*p* value	Beta value	*p* value	Beta value	*p* value	Beta value	*p* value
**Antidepressant use**	0.16	0.34	0.07	0.69	0.15	0.35	0.04	0.80	0.39	0.05	0.28	0.18	0.53	<0.001	0.36	0.06	0.8	<0.001	0.55	0.01

Abbreviations: HDL-C, high-density lipoprotein-cholesterol; MetS, metabolic syndrome.

Model 1: Adjusted for: age, sex, race, education, family income-to-poverty ratio, insurance, marital status, physical activity, BMI, smoking status, alcohol consumption.

Model 2: Additionally adjusted for depressive symptoms.

Table S8. Odds ratios for individual and clustered MetS components by depressive symptoms in men and women.[a]

	Individual MetS components									
	Hypertension		Raised triglyceride		Reduced HDL-C		Central obesity		Elevated blood glucose	
	Model 1	Model 2	Model 1	Model 2	Model 1	Model 2	Model 1	Model 2	Model 1	Model 2
**Men**										
Depressive symptoms										
Normal	1 (ref)	1 (ref)	1 (ref)	1 (ref)	1 (ref)	1 (ref)	1 (ref)	1 (ref)	1 (ref)	1 (ref)
Mild	1.16 (0.92-1.47)	1.11 (0.88-1.40)	1.25 (0.99-1.58)	1.18 (0.93-1.49)	1.19 (0.94-1.50)	1.16 (0.92-1.46)	1.04 (0.75-1.43)	1.04 (0.76-1.42)	1.10 (0.83-1.45)	1.08 (0.82-1.44)
Moderate	1.06 (0.75-1.50)	0.96 (0.67-1.37)	**1.58 (1.10-2.27)**	1.38 (0.94-2.01)	1.27 (0.86-1.86)	1.21 (0.81-1.80)	1.31 (0.68-2.52)	1.31 (0.68-2.52)	1.02 (0.72-1.43)	0.98 (0.70-1.37)
Severe	1.65 (0.93-2.93)	1.41 (0.81-2.45)	**2.10 (1.29-3.4)**	**1.74 (1.04-2.92)**	1.27 (0.77-2.09)	1.18 (0.69-2.04)	0.77 (0.39-1.53)	0.78 (0.38-1.59)	1.15 (0.68-1.94)	1.09 (0.64-1.85)
**Women**										
Depressive symptoms										
Normal	1 (ref)	1 (ref)	1 (ref)	1 (ref)	1 (ref)	1 (ref)	1 (ref)	1 (ref)	1 (ref)	1 (ref)
Mild	1.07 (0.87-1.32)	1.03 (0.83-1.29)	**1.31 (1.06-1.63)**	**1.28 (1.03-1.58)**	0.95 (0.78-1.15)	0.96 (0.79-1.16)	1.09 (0.81-1.47)	1.03 (0.76-1.41)	1.12 (0.94-1.34)	1.13 (0.94-1.37)
Moderate	**1.67 (1.23-2.28)**	**1.55 (1.09-2.21)**	**1.70 (1.20-2.42)**	**1.61 (1.13-2.29)**	1.15 (0.87-1.52)	1.17 (0.87-1.56)	**2.06 (1.17-3.62)**	**1.94 (1.08-3.46)**	**1.59 (1.11-2.28)**	**1.62 (1.13-2.32)**
Severe	1.01 (0.63-1.63)	0.94 (0.57-1.53)	1.08 (0.70-1.67)	1.02 (0.65-1.60)	1.04 (0.74-1.46)	1.06 (0.75-1.50)	1.12 (0.63-2.01)	1.03 (0.57-1.87)	1.46 (1.00-2.13)	1.48 (1.00-2.20)

	Clustered number of MetS components									
	One (vs None)		Two (vs None)		Three (vs None)		Four (vs None)		Five (vs None)	
	Model 1	Model 2	Model 1	Model 2	Model 1	Model 2	Model 1	Model 2	Model 1	Model 2
**Men**										
Depressive symptoms										
Normal	1 (ref)	1 (ref)	1 (ref)	1 (ref)	1 (ref)	1 (ref)	1 (ref)	1 (ref)	1 (ref)	1 (ref)
Mild	1.24 (0.88-1.73)	1.24 (0.89-1.75)	**1.59 (1.03-2.44)**	**1.58 (1.02-2.46)**	1.21 (0.81-1.82)	1.14 (0.76-1.71)	1.53 (0.95-2.49)	1.45 (0.89-2.35)	**2.36 (1.26-4.43)**	**2.06 (1.11-3.85)**
Moderate	1.49 (0.77-2.90)	1.53 (0.79-2.93)	1.83 (0.90-3.72)	1.81 (0.92-3.58)	1.34 (0.64-2.78)	1.18 (0.58-2.39)	1.16 (0.50-2.70)	1.03 (0.44-2.41)	**4.80 (1.92-11.98)**	**3.75 (1.45-9.72)**
Severe	**2.70 (1.29-5.64)**	**2.77 (1.39-5.52)**	**2.56 (1.15-5.73)**	**2.47 (1.16-5.25)**	2.58 (0.93-7.22)	2.09 (0.79-5.53)	**4.66 (1.94-11.19)**	**3.86 (1.62-9.20)**	**5.83 (1.61-21.20)**	**4.2 (1.19-14.76)**
**Women**										
Depressive symptoms										
Normal	1 (ref)	1 (ref)	1 (ref)	1 (ref)	1 (ref)	1 (ref)	1 (ref)	1 (ref)	1 (ref)	1 (ref)
Mild	1.07 (0.78-1.45)	1.05 (0.78-1.41)	0.96 (0.71-1.29)	0.95 (0.70-1.30)	1.20 (0.86-1.68)	1.19 (0.84-1.68)	1.17 (0.77-1.77)	1.13 (0.73-1.74)	1.44 (0.89-2.32)	1.37 (0.85-2.19)
Moderate	1.50 (0.77-2.91)	1.47 (0.78-2.79)	1.54 (0.84-2.82)	1.55 (0.84-2.86)	**2.24 (1.18-4.25)**	**2.22 (1.15-4.28)**	**3.64 (1.65-8.00)**	**3.40 (1.48-7.78)**	**4.24 (2.05-8.81)**	**3.87 (1.85-8.13)**
Severe	1.40 (0.71-2.75)	1.36 (0.68-2.73)	1.29 (0.66-2.52)	1.29 (0.64-2.57)	1.47 (0.74-2.92)	1.43 (0.70-2.92)	1.73 (0.81-3.70)	1.6 (0.73-3.54)	1.69 (0.71-4.03)	1.53 (0.62-3.77)

Abbreviations: HDL-C, high-density lipoprotein-cholesterol; MetS, metabolic syndrome.
Model 1: Adjusted for: age, race, education, family income ratio, insurance, marital status, physical activity, BMI, smoking status, alcohol consumption.
Model 2: Additionally adjusted for antidepressant use.
[a] The values of odds ratio (95%CI) with boldface indicates statistical significance (P<0.05).

Table S9. Odds ratios for individual and clustered MetS components by antidepressant use in men and women.[a]

	Individual MetS components									
	Hypertension		Raised triglyceride		Reduced HDL-C		Central obesity		Elevated blood glucose	
	Model 1	Model 2	Model 1	Model 2	Model 1	Model 2	Model 1	Model 2	Model 1	Model 2
**Men**										
Antidepressant use (vs non-users)	**1.66 (1.21-2.26)**	**1.59 (1.17-2.17)**	**1.93 (1.43-2.61)**	**1.76 (1.28-2.43)**	1.30 (0.99-1.70)	1.23 (0.92-1.65)	0.99 (0.70-1.40)	0.99 (0.69-1.42)	1.20 (0.88-1.63)	1.18 (0.86-1.62)
**Women**										
Antidepressant use (vs non-users)	**1.33 (1.05-1.67)**	1.27 (0.97-1.65)	**1.29 (1.03-1.62)**	1.19 (0.95-1.50)	0.95 (0.77-1.19)	0.94 (0.75-1.18)	1.39 (0.99-1.94)	1.32 (0.93-1.88)	1.02 (0.82-1.27)	0.95 (0.75-1.19)

	Clustered number of MetS components									
	One (vs None)		Two (vs None)		Three (vs None)		Four (vs None)		Five (vs None)	
	Model 1	Model 2	Model 1	Model 2	Model 1	Model 2	Model 1	Model 2	Model 1	Model 2
**Men**										
Antidepressant use (vs non-users)	1.07 (0.58-1.96)	0.90 (0.50-1.62)	1.33 (0.69-2.54)	1.08 (0.58-2.02)	**2.10 (1.11-3.97)**	**1.88 (1.02-3.46)**	**2.14 (1.12-4.07)**	1.79 (0.96-3.34)	**3.82 (1.80-8.08)**	**2.69 (1.27-5.71)**
**Women**										
Antidepressant use (vs non-users)	1.14 (0.76-1.72)	1.09 (0.74-1.61)	1.03 (0.68-1.56)	1.00 (0.66-1.53)	1.17 (0.70-1.95)	1.06 (0.63-1.80)	1.46 (0.92-2.32)	1.26 (0.77-2.08)	1.59 (0.97-2.63)	1.34 (0.80-2.25)

Abbreviations: HDL-C, high-density lipoprotein-cholesterol; MetS, metabolic syndrome.

Model 1: Adjusted for: age, race, education, family income ratio, insurance, marital status, physical activity, BMI, smoking status, alcohol consumption

Model 2: Additionally adjusted for depressive symptoms.

[a] The values of odds ratio (95%CI) with boldface indicates statistical significance (P<0.05).

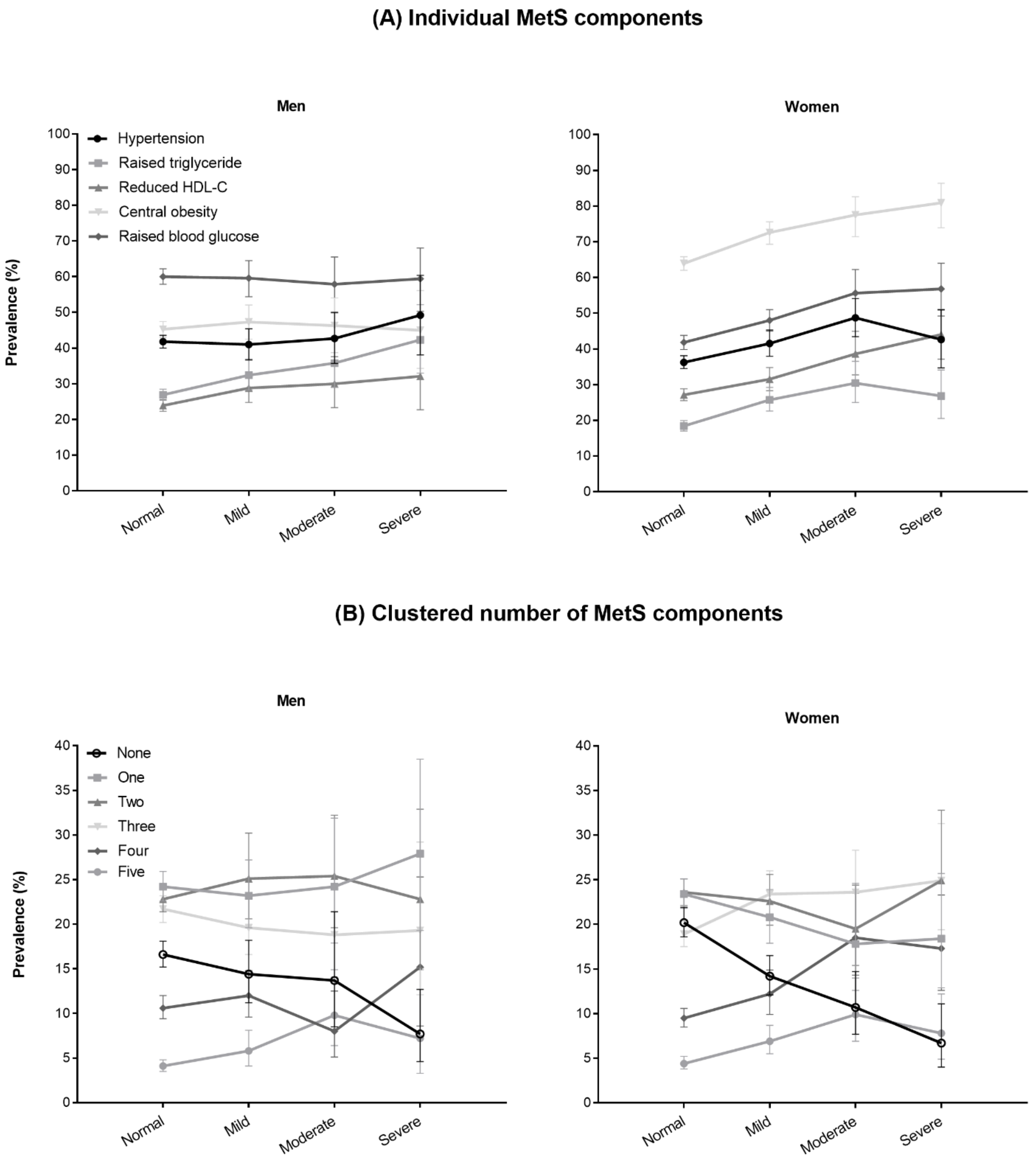

Figure S1. Prevalence of individual (A) and clustered (B) MetS components by depressive symptoms in men and women.

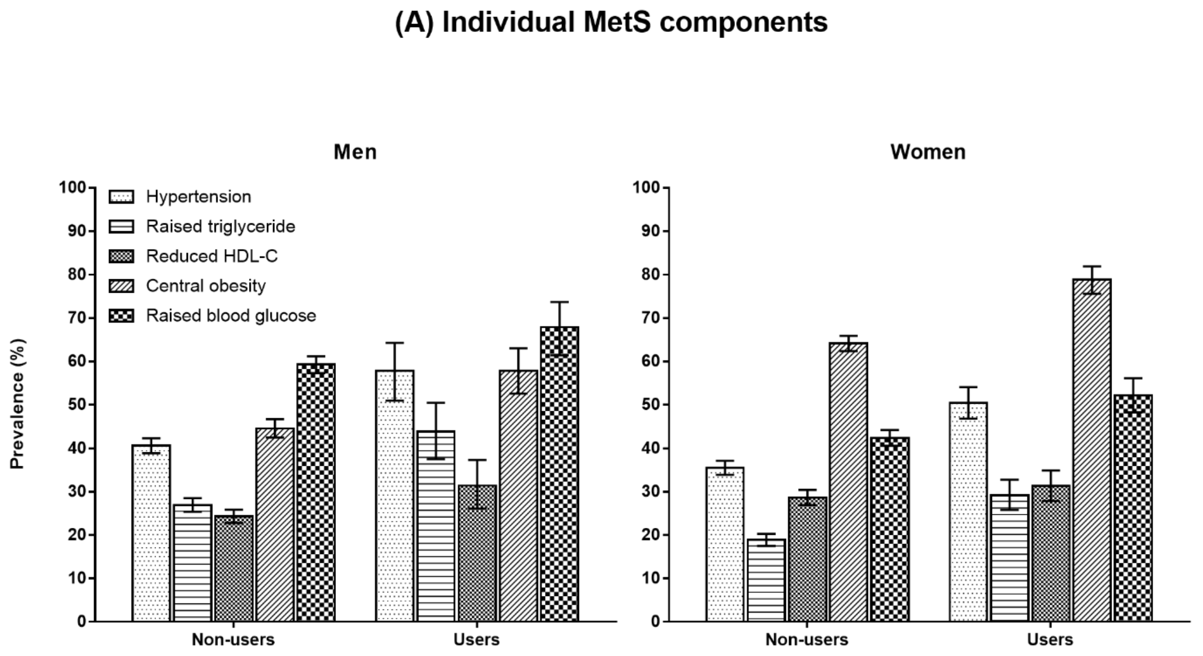

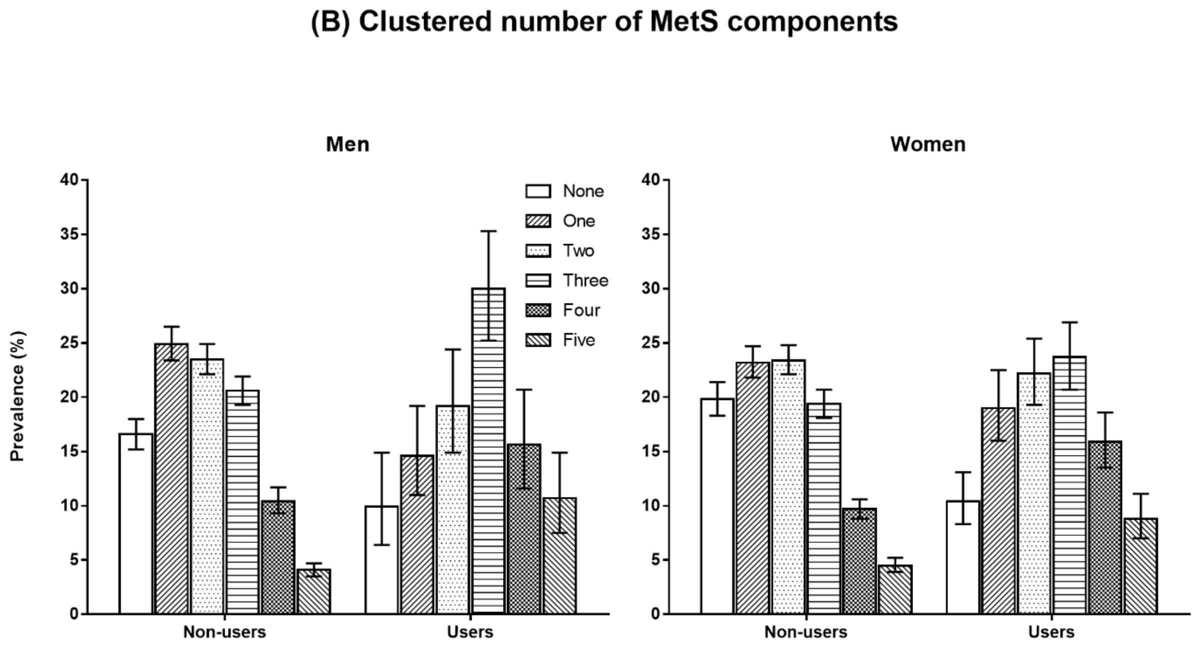

Figure S2. Prevalence of individual (A) and clustered (B) MetS components by antidepression use in men and women.
